# RNA *N* ^6^‐Methyladenosine‐Binding Protein YTHDFs Redundantly Attenuate Cancer Immunity by Downregulating IFN‐γ Signaling in Gastric Cancer

**DOI:** 10.1002/advs.202410806

**Published:** 2024-11-25

**Authors:** Dongjun Jang, Chanwoong Hwa, Seoyeon Kim, Jaeik Oh, Seungjae Shin, Soo‐Jin Lee, Jiwon Kim, Sang Eun Lee, Yoojin Yang, Dohee Kim, Seoho Lee, Hae Rim Jung, Yumi Oh, Kyunggon Kim, Hye Seung Lee, Joon‐Yong An, Sung‐Yup Cho

**Affiliations:** ^1^ Department of Biomedical Sciences Seoul National University College of Medicine Seoul 03080 South Korea; ^2^ L‐HOPE Program for Community‐Based Total Learning Health Systems Korea University Seoul 02841 South Korea; ^3^ Department of Integrated Biomedical and Life Science Korea University Seoul 02841 South Korea; ^4^ Department of Translational Medicine Seoul National University College of Medicine Seoul 03080 South Korea; ^5^ Medical Research Center, Genomic Medicine Institute Seoul National University College of Medicine Seoul 03080 South Korea; ^6^ Department of Biomedical Sciences University of Ulsan College of Medicine Seoul 05505 South Korea; ^7^ Department of Pathology Seoul National University College of Medicine Seoul 03080 South Korea; ^8^ Cancer Research Institute Seoul National University Seoul 03080 South Korea; ^9^ School of Biosystem and Biomedical Science College of Health Science Korea University Seoul 02841 South Korea

**Keywords:** cancer immunity, gastric cancer, interferon‐γ signaling, N^6^‐methyladenosine, YTHDF proteins

## Abstract

Immunotherapy holds potential as a treatment for gastric cancer (GC), though immune checkpoint inhibitor (ICI) resistance remains an obstacle. One resistance mechanism involves defects in interferon‐γ (IFN‐γ) signaling, in which IFN‐γ is linked to improved responsiveness to ICIs. Herein, the roles of RNA *N^6^*‐methyladenosine (m6A) modifications in regulation of IFN‐γ signaling and the responsiveness to ICIs are unveiled. The m6A‐binding protein YTH *N* ^6^‐methyladenosine RNA‐binding protein F1 (YTHDF1) is overexpressed in GC tissues, correlating with the suppression of cancer immunity and poorer survival rates. YTHDF1 overexpression impaired the responsiveness to IFN‐γ in GC cells, and knockdown studies indicated the redundant effects of YTHDF2 and YTHDF3 with YTHDF1 in IFN‐γ responsiveness. RNA immunoprecipitation sequencing revealed YTHDFs directly target interferon regulatory factor 1 (IRF1) mRNA, a master regulator of IFN‐γ signaling, leading to reduced RNA stability and consequent downregulation of IFN‐γ signaling. Furthermore, in mouse syngeneic tumor models, Ythdf1 depletion in cancer cells resulted in reduced tumor growth and increased tumor‐infiltrating lymphocytes, which are attributed to the augmentation of IFN‐γ signaling. Collectively, these findings highlight how YTHDFs modulate cancer immunity by influencing IFN‐γ signaling through IRF1 regulation, suggesting their viability as therapeutic targets in cancer immunotherapy.

## Graphical abstract

The RNA *N* ^6^‐methyladenosine‐binding protein YTHDF1 is overexpressed in gastric cancer tissues and associates with the suppression of cancer immunity. YTHDF1, along with YTHDF2 and YTHDF3, redundantly suppresses the IFN‐γ response by destabilizing IRF1 mRNA, suggesting that these proteins can serve as viable therapeutic targets in cancer immunotherapy.

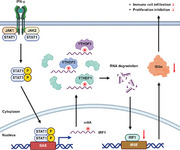

## 1 Introduction

Gastric cancer (GC) is a major public health problem, particularly in East Asia, and ranks fifth in cancer incidence and fifth in cancer‐related mortality worldwide. The main treatment options for advanced GC are combinations of chemotherapy, targeted therapy, and immunotherapy.^[^ ^1^ ^]^ Notably, immunotherapy that blocks cytotoxic T lymphocyte‐related antigen 4 (CTLA‐4) or the programmed apoptotic protein 1 (PD‐1)/programmed death‐ligand 1 (PD‐L1) pathway with antibodies has garnered attention due to its potential for achieving complete remission.^[^ ^2^ ^]^ Although immunotherapy has been successfully applied for the clinical treatment of GC, several clinical trials have reported that the efficacy of immune checkpoint blockers as monotherapy in later lines is limited, and a substantial proportion of patients demonstrate unresponsiveness to immunotherapy.^[^ ^3^ ^]^ To overcome this challenge, it is crucial to gain a comprehensive understanding of the molecular mechanisms underlying resistance.

One of the resistance mechanisms involves defects in neoantigen processing and presentation through the interferon‐gamma (IFN‐γ) signaling cascade.^[^ ^4^ ^]^ In addition, it has been suggested that patients expressing high levels of IFN‐γ are more responsive to immune checkpoint inhibitors (ICIs) compared with patients with lower levels.^[^ ^4^ ^]^ IFN‐γ activates the Janus kinase (JAK)–signal transducer and activator of transcription (STAT) pathway, leading to the translocation of STAT1 dimers to the nucleus. Once in the nucleus, STAT1 acts as a transcription factor, facilitating the expression of IFN‐γ‐related genes, including interferon regulatory factor 1 (IRF1).^[^ ^5^ ^]^ IRF1 is a master regulator of the IFN‐γ‐mediated inflammatory response and antigen presentation and demonstrates a strong association with improved responsiveness to cancer treatment.^[^ ^6^ ^]^ Therefore, investigations of IFN‐γ/JAK–STAT/IRF1 axis regulation are required to gain insights into the molecular mechanisms associated with responsiveness in cancer immunotherapy.

Recent reports suggest that *N* ^6^‐methyladenosine (m6A) modification plays crucial roles in regulating RNA metabolism and function, influencing both carcinogenesis and immune responses.^[^ ^7^ ^]^ The m6A modification is a chemical modification of adenosine in RNA and the most prevalent RNA modification observed across various eukaryotes.^[^ ^8^ ^]^ This modification is catalyzed by a large methyltransferase complex containing methyltransferase‐like 3 (METTL3), METTL14, and Wilms tumor 1 associated protein (WTAP) (m6A writers) and is removed by fat mass and obesity‐associated protein (FTO) and AlkB homolog 5 (ALKBH5) (m6A erasers). The m6A‐modified transcripts are recognized by m6A readers, such as YTH *N* ^6^‐methyladenosine RNA‐binding protein F (YTHDF)1–3, YTH *N* ^6^‐methyladenosine RNA‐binding protein C (YTHDC)1–2, and insulin‐like growth factor 2 mRNA‐binding protein (IGF2BP)1–3, which modulate RNA splicing, nuclear export, translation, and degradation.^[^ ^9^ ^]^ RNA m6A‐binding proteins play substantial roles in cancer development and are highly associated with patient prognosis in several cancer types.^[^ ^10^ ^]^ Among m6A readers, it has been suggested that YTHDF1 promotes mRNA translation in conjunction with translation initiation factors, YTHDF2 enhances mRNA degradation, and YTHDF3 is involved in both mRNA translation and degradation processes.^[^ ^11^ ^]^ However, YTHDF1–3 have also been recognized to bind the same mRNA m6A sites and enhance mRNA degradation with the carbon catabolite repression–negative on TATAless (CCR4–NOT) complex.^[^ ^12^ ^]^ Regardless, YTHDF proteins have been implicated in numerous biological processes and disease phenotypes through their regulation of post‐transcriptional gene expression.

In this study, we suggested the suppressive effect of YTHDFs on cancer immunity, particularly in modulating the responsiveness to IFN‐γ in cancer cells. Downregulation of YTHDF1 enhanced IFN‐γ signaling in both cell line and animal models, and concurrent downregulation of YTHDF2 or YTHDF3 exhibited a synergistic effect with YTHDF1 suppression. Through transcriptomic and proteomic analyses, we identified IRF1 as a direct regulatory target of YTHDFs in IFN‐γ signaling. These findings highlight that YTHDFs are potential therapeutic targets in cancer immunotherapy.

## 2 Results

### 2.1 YTHDF1 Expression is Associated with a Decreased Immune Response in GC

**Figure 1:**
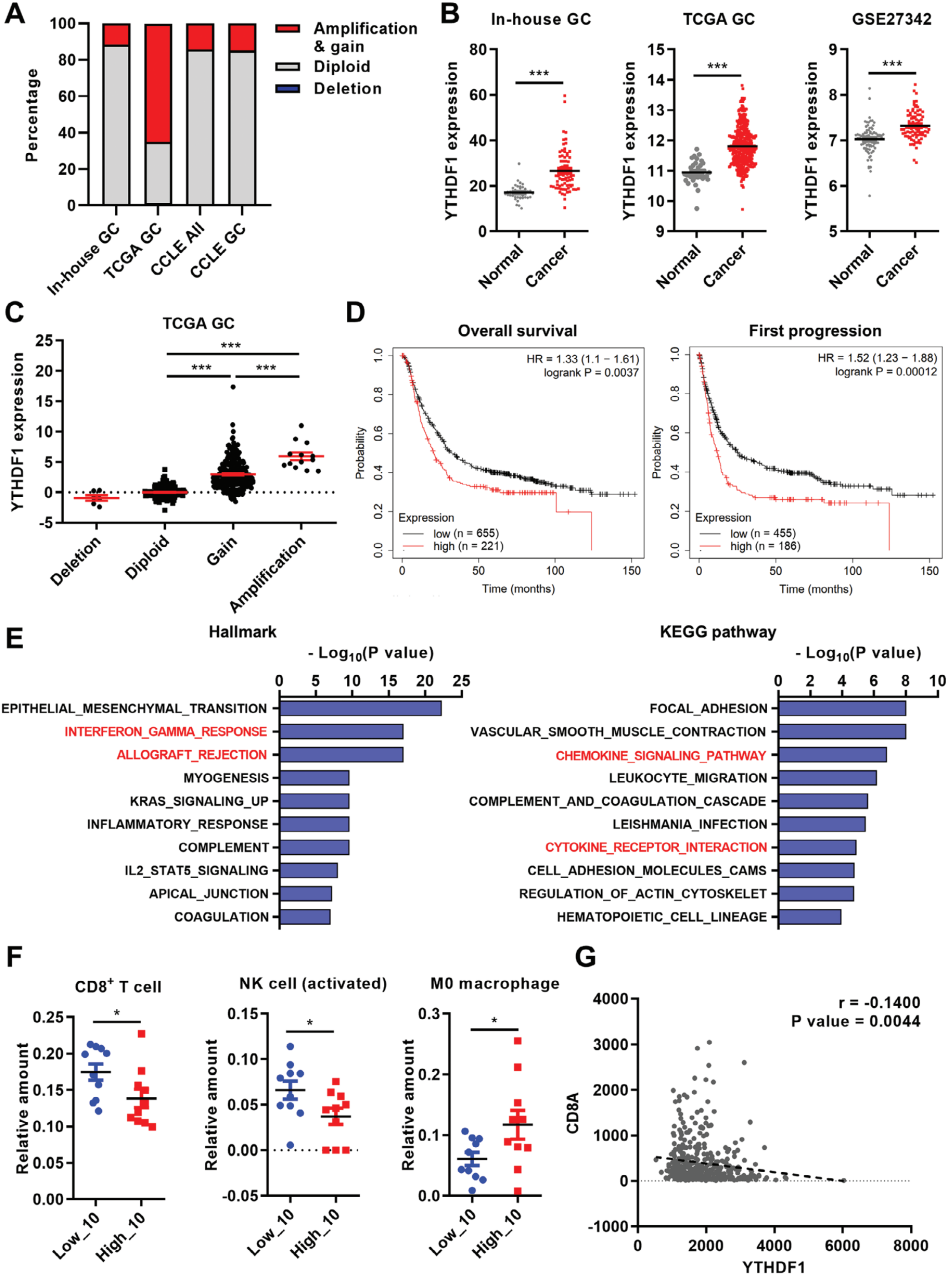
YTHDF1 is overexpressed in gastric cancer (GC) and associated with an immunosuppressive phenotype. A) Proportion of cancer samples exhibiting DNA copy number alterations in YTHDF1. The copy numbers of YTHDF1 gene were analyzed by array comparative genomic hybridization (aCGH) for in‐house GC samples (*n* = 103) or by GISTIC 2.0 using RNA sequencing data for The Cancer Genome Atlas (TCGA) GC (*n* = 410) and Cancer Cell Line Encyclopedia (CCLE; *n* = 916 for all cell lines and *n* = 47 for GC cell lines). B) YTHDF1 mRNA expressions in GC cohort. Gene expression data were analyzed from in‐house GC data (normal, *n* = 42; cancer, *n* = 76), The Cancer Genome Atlas (TCGA) GC cohort (normal, *n* = 36; cancer, *n* = 414), and microarray data of GC tissues from Gene Expression Omnibus (GEO) (GSE27342; normal, *n* = 80; cancer, *n* = 80). *p* values were calculated using unpaired *t*‐test (left and middle) or paired *t*‐test (right; ***p < 0.001). C) The mRNA expression levels of YTHDF1 according to DNA copy number alterations in TCGA GC cohort. Samples were categorized into four groups based on DNA copy numbers of YTHDF1 genes, and the mRNA expressions of YTHDF1 in each category were compared with the diploid group. *p* values were calculated by using one‐way ANOVA (***p < 0.001). D) Kaplan–Meier analysis for the overall survival (left) and first progression survival (right) based on YTHDF1 mRNA expression in GC. Red and blue lines stand for samples with high and low YTHDF1 expression, respectively. Each hazard ratio with 95% confidence interval and *p* value, determined by log rank test, is shown. E) Gene set analysis of 642 genes negatively correlated with YTHDF1 (*p* < 0.0001, r < −0.3) in TCGA GC cohort. The top ten hallmark pathway (left) and KEGG pathway gene sets (right) that were significantly enriched in the 642 genes are shown. F) The distribution patterns of CD8^+^ T cells, activated NK cells, and M0 macrophages according to YTHDF1 mRNA expressions. Relative amounts of each immune cell type analyzed by CIBERSORT using ten samples with the highest and lowest expression of YTHDF1 in TCGA GC cohort were demonstrated. *p* values were calculated using unpaired *t*‐test (*p < 0.05). G) Scatter plot displaying the correlation of YTHDF1 with CD8A mRNA expression in the TCGA GC cohort. Spearman's correlation coefficient (r) and *p* value of correlation is shown.

In a previous study, we explored genomic alterations in 103 normal‐matched GC tissues using an array comparative genomic hybridization (aCGH) platform.^[^ ^13^ ^]^ From this dataset, we found that the YTHDF1 copy number increased in 12 out of 103 cases (11.7%; **Figure** **1**A; Figure S1A, Supporting Information). We also detected the amplification or gain of the YTHDF1 gene in a substantial proportion of samples from The Cancer Genome Atlas (TCGA) GC cohort (*n* = 410, 65.0%) and the Cancer Cell Line Encyclopedia (CCLE; all cell lines, *n* = 916, 14.2%; GC cell lines, *n* = 47, 14.9%) (Figure 1A). Accordingly, YTHDF1 mRNA expression levels were significantly upregulated in GC tissues compared with normal gastric tissues (Figure 1B). A positive correlation was observed between DNA copy number increase and the upregulation of YTHDF1 RNA expression (Figure 1C; Figure S1B, Supporting Information). Patients with high YTHDF1 expression levels demonstrated significantly worse overall survival and first‐progression survival outcomes in the TCGA GC cohort (Figure 1D).

To understand the biological implications of YTHDF1 upregulation in GC, we identified genes that exhibited positive correlations (Spearman's correlation r > 0.3, *p* < 0.0001, *n* = 1004 genes) or negative correlations (Spearman's correlation r < −0.3, *p* < 0.0001, *n* = 642 genes) with YTHDF1 expression levels in the TCGA GC cohort (Figure S1C, Supporting Information). Pathway analyses of positive correlated genes revealed enrichment in several cell cycle‐related gene sets, including “G2M checkpoint” and “E2F targets” from the hallmark gene sets (https://www.gsea‐msigdb.org/gsea/msigdb/collections.jsp) and “Cell cycle” from the Kyoto Encyclopedia of Genes and Genomes (KEGG) pathway gene sets (https://www.genome.jp/kegg/genes.html) (Figure S1D, Supporting Information). Additional pathway analyses revealed that negative correlated genes were enriched in several cancer immunity‐related gene sets, including “Interferon‐gamma response,” and “Allograft rejection” from the hallmark gene sets and “Chemokine signaling pathway” and “Cytokine receptor interaction” from the KEGG pathway gene sets (Figure 1E). Consistently, a cell type proportion estimation analysis based on TCGA RNA sequencing (RNA‐seq) data revealed decreased abundances of CD8^+^ T cells and natural killer (NK) cells, along with an increase in M0 macrophages, in the high YTHDF1‐expressing group (Figure 1F).^[^ ^14^ ^]^ We also observed negative correlations between YTHDF1 expression and the expression levels of CD8 subunit alpha (CD8A), granzyme A (GZMA), granzyme B (GZMB), CD69, interleukin 2 receptor subunit alpha (IL2RA), CD40L, PD‐1, tumor necrosis factor receptor superfamily member 9 (TNFRSF9) and CD28, which are activated cytotoxic T‐cell markers (Figure 1G; Figure S2, Supporting Information). These data indicated a potential role for YTHDF1 in immune evasion mechanisms.

### 2.2 YTHDF1 Overexpression Reduces the IFN‐γ Response in GC Cells

**Figure 2:**
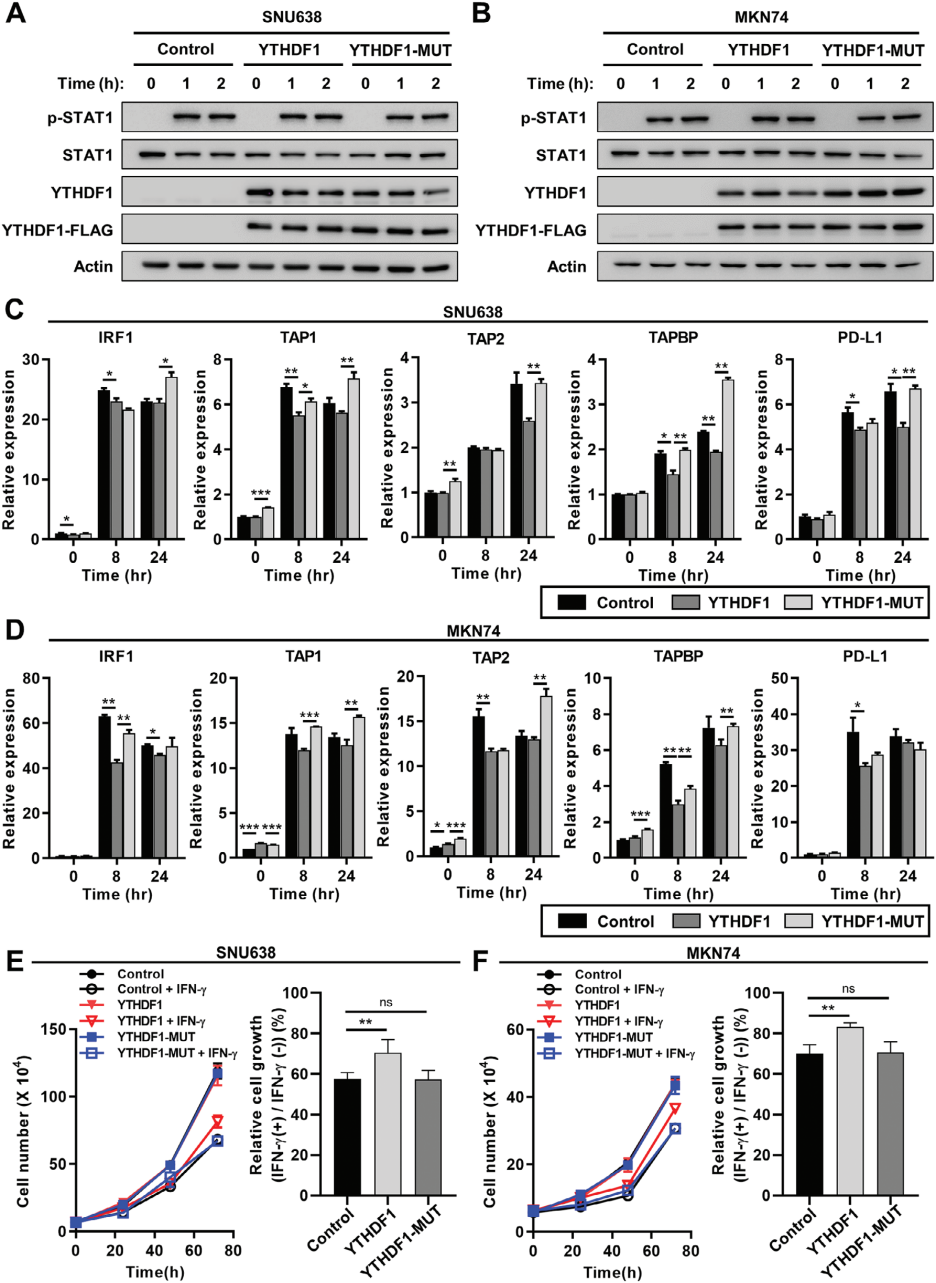
YTHDF1 overexpression downregulates IFN‐γ response in GC cells. A,B) Phosphorylation of STAT1 in wild‐type YTHDF1 or m6A‐binding defective mutant YTHDF1 (YTHDF1‐MUT)‐overexpressed GC cells. After serum starvation for 24 h, SNU638 (A) and MKN74 (B) cells were treated with 1 ng mL^−1^ IFNγ for indicated time. The phosphorylation levels of STAT1 were evaluated by western blotting. C,D) The expression of IFNγ‐responsive genes in wild‐type YTHDF1 or YTHDF1‐MUT‐overexpreesed GC cells. Wild‐type YTHDF1 or YTHDF1‐MUT was overexpressed for 24 h in SNU638 (C) and MKN74 (D) cells. After serum starvation for 24 h, cells were treated with 10 ng mL^−1^ IFNγ, and the mRNA expression levels of IFNγ‐responsive genes were estimated by qPCR relative to the levels of GAPDH (*n* = 3) at indicated time points. Relative values are compared to 0 h in control. *p* values were calculated using one‐way ANOVA (*p < 0.05, **p < 0.01, ***p < 0.001). E,F) Suppression of cell proliferation by IFNγ treatment in wild‐type YTHDF1 or YTHDF1‐MUT‐overexpressed GC cells. After serum starvation for 24 h, SNU638 (E) and MKN74 (F) cells were treated with 10 ng mL^−1^ IFNγ for indicated time points and the cell numbers were estimated by trypan blue staining assay (left). Relative cell growth is estimated compared to each IFN‐γ‐untreated control in 72 h (right). *p* values were calculated using one‐way ANOVA (**p < 0.01).

Because YTHDF1 expression was inversely correlated with genes related to “Interferon‐gamma response” (Figure 1E), we investigated the effect of YTHDF1 overexpression on IFN‐γ signaling. Upon receptor binding, IFN‐γ triggers the phosphorylation and nuclear translocation of STAT1, leading to the expression of interferon‐stimulated genes. This cascade of events ultimately leads to a reduction in cell growth and an enhancement in antigen processing.^[^ ^15^ ^]^ The overexpression of FLAG‐tagged YTHDF1 and its mutant form (YTHDF1‐MUT; K395A and Y397A), which fails to bind to m6A,^[^ ^16^ ^]^ had little effect on the phosphorylation levels of STAT1 (**Figure** **2**A,B). However, YTHDF1 overexpression suppressed IFN‐γ‐mediated expression of IFN‐γ‐regulated genes, including transporter associated with antigen processing 1 (TAP1), TAP binding protein (TAPBP), and PD‐L1 in SNU638 cells, and IRF1, TAP1, and TAPBP in MKN74 cells (Figure 2C,D). In contrast, the mutant YTHDF1 failed to elicit any significant effects on these genes (Figure 2C,D), indicating that YTHDF1 suppresses the IFN‐γ response by downregulating IFN‐γ‐induced mRNA gene expression downstream of STAT1. Furthermore, YTHDF1 attenuated the suppressive effect of IFN‐γ on cell proliferation, whereas the mutant form of YTHDF1 exhibited no discernible effect (Figure 2E,F).

**Figure 3:**
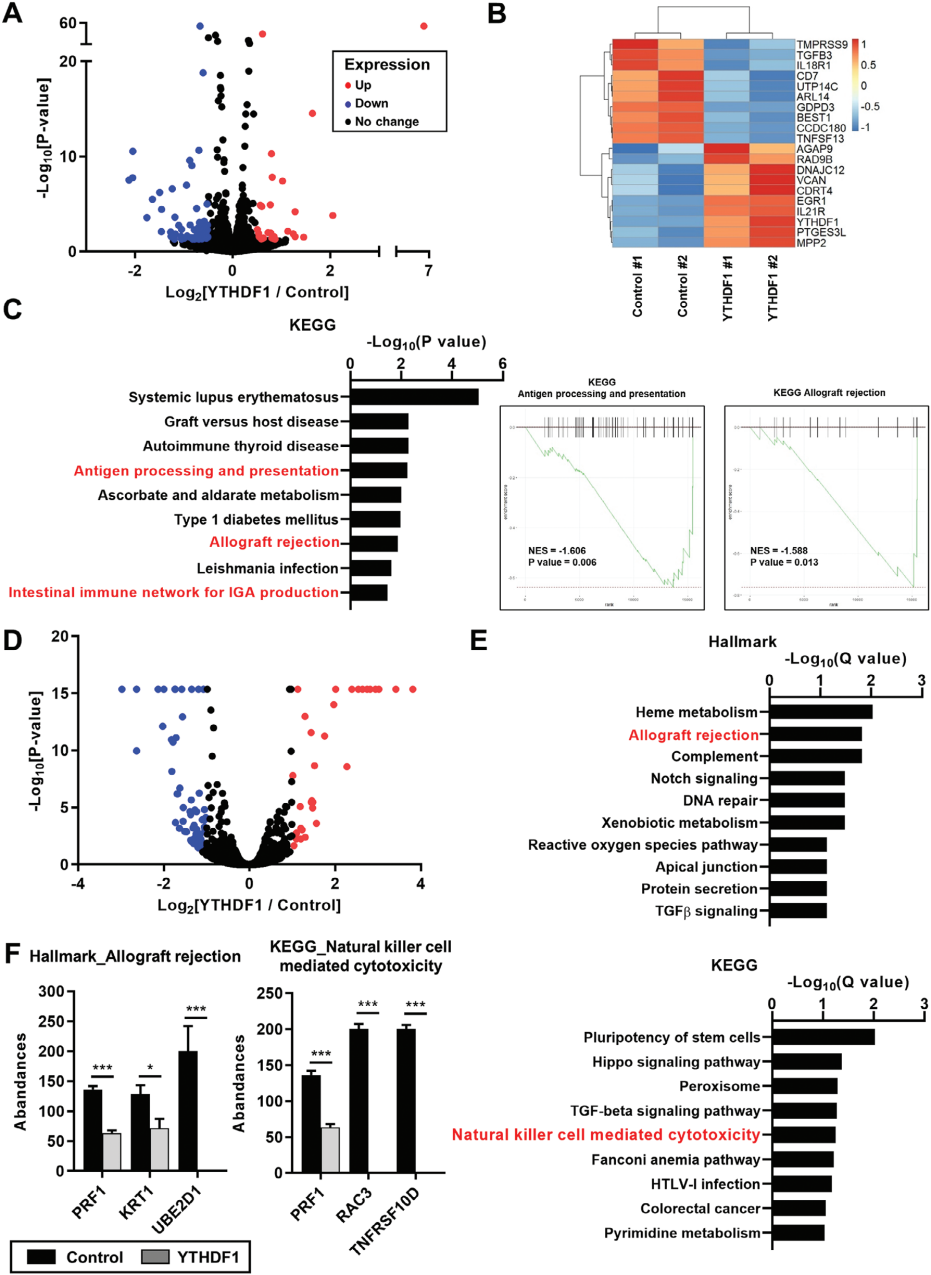
Transcriptomic and proteomic profiles suggest YTHDF1‐mediated suppression of cancer immunity‐related gene sets in the presence of IFNγ treatment. A) Volcano plot illustrating the relative mRNA expression levels of genes in RNA sequencing analysis of control vector or YTHDF1‐overexpressed SNU638 cells (*n* = 2). After treatment of 10 ng mL^−1^ IFNγ for 24 h, transcriptomic analysis was performed using RNA sequencing. Red and blue dots indicate upregulated and downregulated genes, respectively (Log_2_[fold change] > 0.5 or < −0.5; *p *value < 0.05). B) Heatmap showing top ten differentially expressed coding genes between control vector and YTHDF1‐overexpressed SNU638 cells (*n* = 2) by RNA‐sequencing. Each column stands for a cell line sample, and each row denotes a gene. Red and blue indicate genes with high and low z‐scores, respectively. C) Gene set enrichment analysis (GSEA) for RNA sequencing from wild‐type and YTHDF1‐overexpressed SNU638 cells in the presence of IFNγ. The left graph shows the top nine significantly depleted KEGG pathway gene sets in YTHDF1‐overexpressed cells. The right graphs show the enrichment plots of representative gene sets that were significantly depleted in YTHDF1‐overexpressed cells. On the x‐axis, genes are ranked from the most upregulated to the most downregulated between YTHDF1‐overexpressed (left end; positively correlated) and control (right end; negatively correlated) cells. The y‐axis shows a running enrichment score for YTHDF1‐overexpression. Enrichment plots demonstrate that “Antigen processing and presentation” and “Allograft rejection” pathways exhibit a negative enrichment associated with YTHDF1 overexpression. D) Volcano plot illustrating the relative protein levels of genes in proteomic analysis for newly synthesized proteins of control vector or YTHDF1‐overexpressed SNU638 cells (*n* = 3). After treatment of 10 ng mL^−1^ IFNγ for 24 h, proteomic analysis for newly synthesized proteins labeled with azidohomoalanine (AHA) was performed. Red and blue dots indicate upregulated and downregulated proteins, respectively (Log_2_[fold change] > 1 or < −1; *p *value < 0.05). E) Gene set analysis for differential expressed proteins (DEPs) by YTHDF1 overexpression. The DEPs downregulated by YTHDF1 overexpression were analyzed using hallmark gene sets (upper) and KEGG pathway gene sets (low). Hallmark gene sets were analyzed by MSigDB (https://www.gsea‐msigdb.org/gsea/msigdb) and KEGG pathway gene sets were analyzed by DAVID (https://david.ncifcrf.gov/). F) Protein abundance of PRF1, KRT1, UBE2D1, RAC3, and TNFRSF10D after YTHDF1 overexpression in IFNγ‐treated SNU638 cells. Protein abundance was measured by mass spectrometry using three independent biological replicates. *p* values were calculated using unpaired *t*‐test (*p < 0.05, ***p < 0.001).

To investigate the effect of YTHDF1 overexpression on IFN‐γ responsiveness at the omics level, we conducted RNA‐seq and mass spectrometry‐based proteomic analyses in the presence of IFN‐γ (Figure S3A, Supporting Information). YTHDF1 overexpression in IFN‐γ‐treated GC cells led to the significant upregulation of 27 genes and the downregulation of 70 genes (│Log_2_[fold change]│ ≥ 0.5 and *p* < 0.05; **Figure** **3**A,B). Gene set enrichment analysis (GSEA) revealed negative correlations between YTHDF1 overexpression and cancer immunity–related gene sets, including “Antigen processing and presentation” and “Allograft rejection” from the KEGG gene set, as well as “Immunoglobulin mediated immune response” from the gene ontology (GO) gene set (Figure 3C; Figure S3B, Supporting Information). In contrast, YTHDF1 overexpression resulted in the enrichment of cell cycle‐related gene sets, such as “G2M checkpoint” and “E2F targets” from the hallmark gene set (Figure S3C, Supporting Information), which is concordant with the inhibitory effect of YTHDF1 on IFN‐γ‐induced cell cycle arrest (Figure 2E,F).

We further investigated the translational consequences of YTHDF1 overexpression by assessing newly synthesized proteins labeled with azidohomoalanine (AHA) in IFN‐γ‐treated GC cells. Mass spectrometry‐based proteomic analyses revealed differentially expressed proteins (DEPs), including 131 DEPs downregulated and 80 DEPs upregulated by YTHDF1 overexpression (│Log_2_[fold change]│ ≥1 and *p* < 0.05; Figure 3D). YTHDF1 overexpression downregulated the pathways associated with cancer immunity, including “Allograft rejection” from the hallmark gene set, “Natural killer mediated cytotoxicity” from the KEGG gene set, and “Positive regulation of NF‐kappa B signaling” from the GO gene set (Figure 3E,F; Figure S3D, Supporting Information). In contrast, YTHDF1 overexpression enhanced the protein synthesis levels of genes associated with “DNA repair” and “Cellular response to DNA damage stimulus” from the GO gene set (Figure S3E, Supporting Information); these genes are associated with increased DNA synthesis. Overall, these data suggest the involvement of YTHDF1 in modulating cancer immunity through the inhibition of the IFN‐γ response.

### 2.3 Depletion of YTHDFs Enhances the IFN‐γ Response in GC Cells

To further elucidate the effect of YTHDF1 on the IFN‐γ response, we generated knockdown cell lines using Cas9 and single‐guide RNAs (sgRNAs) targeting YTHDF1 in GC cells (Figure S4A,B, Supporting Information). Depletion of YTHDF1 in SNU668 and MKN1 cells had little effect on the IFN‐γ‐responsive phosphorylation of STAT1 (Figure S4A,B, Supporting Information). In addition, knockdown of YTHDF1 demonstrated modest enhancements in IFN‐γ‐induced gene expression, with increases observed in IRF1 and TAPBP in MKN1 cells and in IRF1, TAP1, TAPBP, and PD‐L1 in SNU668 cells at select time points, along with modest growth inhibition (Figure S4C–F, Supporting Information). One possible explanation for this modest effect is the functional redundancy among YTHDF paralogs and the potential compensatory effects of other YTHDFs.

Recent studies suggested that YTHDF proteins work together to regulate the degradation of m6A‐modified mRNAs, and depleting all three YTHDF proteins resulted in the significant stabilization of m6A‐modified mRNAs.^[^ ^12^ ^]^ To elucidate the roles of YTHDF proteins in GC, we performed GSEA analyses on TCGA samples to identify the top 10% and bottom 10% of changes in mRNA expression associated with YTHDF. Interestingly, YTHDF1–3 all showed negative correlations with “Interferon‐gamma response” gene sets (Figure S5A–C, Supporting Information). In addition, distribution of the normalized enrichment score (NES) based on YTHDF expression levels in hallmark gene sets revealed positive correlations with each other (Figure S5D, Supporting Information). These results support the redundant roles of YTHDF proteins in regulating the cancer immune response in GC.

**Figure 4:**
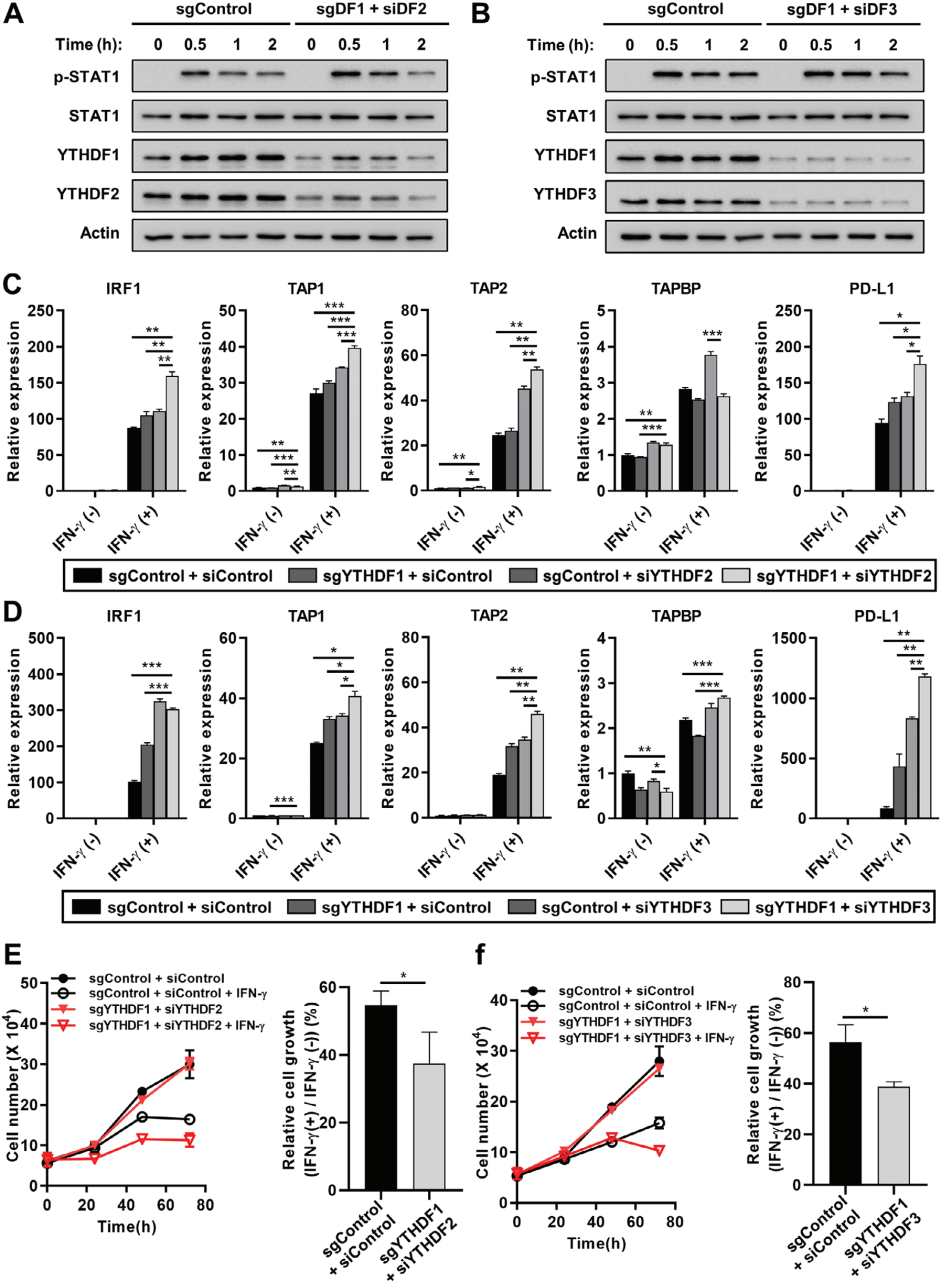
The knockdown of YTHDFs synergistically enhances the IFN‐γ response in GC cells. A,B) Phosphorylation of STAT1 in YTHDF1‐3 knockdown MKN1 cells. YTHDF1 was stably knocked down using the CRISPR/Cas9 method (sgDF1). Then, these cells were transfected with either siRNA targeting YTHDF2 (A) or siRNA targeting YTHDF3 (B) to further downregulate YTHDF2 or YTHDF3 expression. After serum starvation for 24 h, cells were treated with 1 ng mL^−1^ IFNγ for indicated time. The phosphorylation levels of STAT1 were evaluated by western blotting. sgControl: control single guide RNA, siControl: control siRNA, sgDF1: single guide RNA for YTHDF1, siDF2: siRNA for YTHDF2, siDF3: siRNA for YTHDF3. C,D) Expression of IFNγ‐responsive genes in YTHDF1‐3 knockdown MKN1 cells. YTHDF1 stably knocked down were transfected with either siRNA targeting YTHDF2 (C) or siRNA targeting YTHDF3 (D) to further downregulate YTHDF2 or YTHDF3 expression. After serum starvation for 24 h, cells were treated with 10 ng mL^−1^ IFNγ and the mRNA expression levels of IFNγ‐responsive genes were estimated by qPCR relative to the levels of GAPDH (*n* = 3) at indicated time points. Relative values are compared to 0 h in control. *p* values were calculated using one‐way ANOVA (*p < 0.05, **p < 0.01, ***p < 0.001). E, F) Suppression of cell proliferation by IFNγ treatment in YTHDF1‐3 knockdown MKN1 cells. YTHDF1 stably knocked down cells were transfected with either siRNA targeting YTHDF2 (E) or siRNA targeting YTHDF3 (F) to further downregulate YTHDF2 or YTHDF3 expression. After serum starvation for 24 h, cells were treated with 10 ng mL^−1^ IFNγ for indicated time points and the cell numbers were estimated by trypan blue staining assay (left). Relative cell growth is estimated compared to each IFN‐γ‐untreated control in 72 h (right). *p* values were calculated using unpaired *t*‐test (*p < 0.05).

To validate this hypothesis, we depleted YTHDF2 or YTHDF3 using small interfering RNA (siRNA) in YTHDF1 knockdown cells and examined the IFN‐γ response. Although additional depletion of YTHDF2 or YTHDF3 had little effect on the IFN‐γ‐induced phosphorylation of STAT1 levels (**Figure** **4A,B**; Figure S6A,B, Supporting Information), depletion of YTHDF2 or YTHDF3 demonstrated an additive enhancement effect with the depletion of YTHDF1 in IFN‐γ‐induced gene expression levels (Figure 4C,D; Figure S6C,D, Supporting Information). In addition, depletion of YTHDF2 or YTHDF3 with YTHDF1 enhanced IFN‐γ‐induced cell proliferation inhibition (Figure 4E,F; Figure S6E,F, Supporting Information). Taken together, these data suggest that YTHDF proteins had redundant effects in downregulating the IFN‐γ response in GC.

### 2.4 YTHDF1 Downregulates IRF1 Expression by Destabilizing IRF1 mRNAs

**Figure 5:**
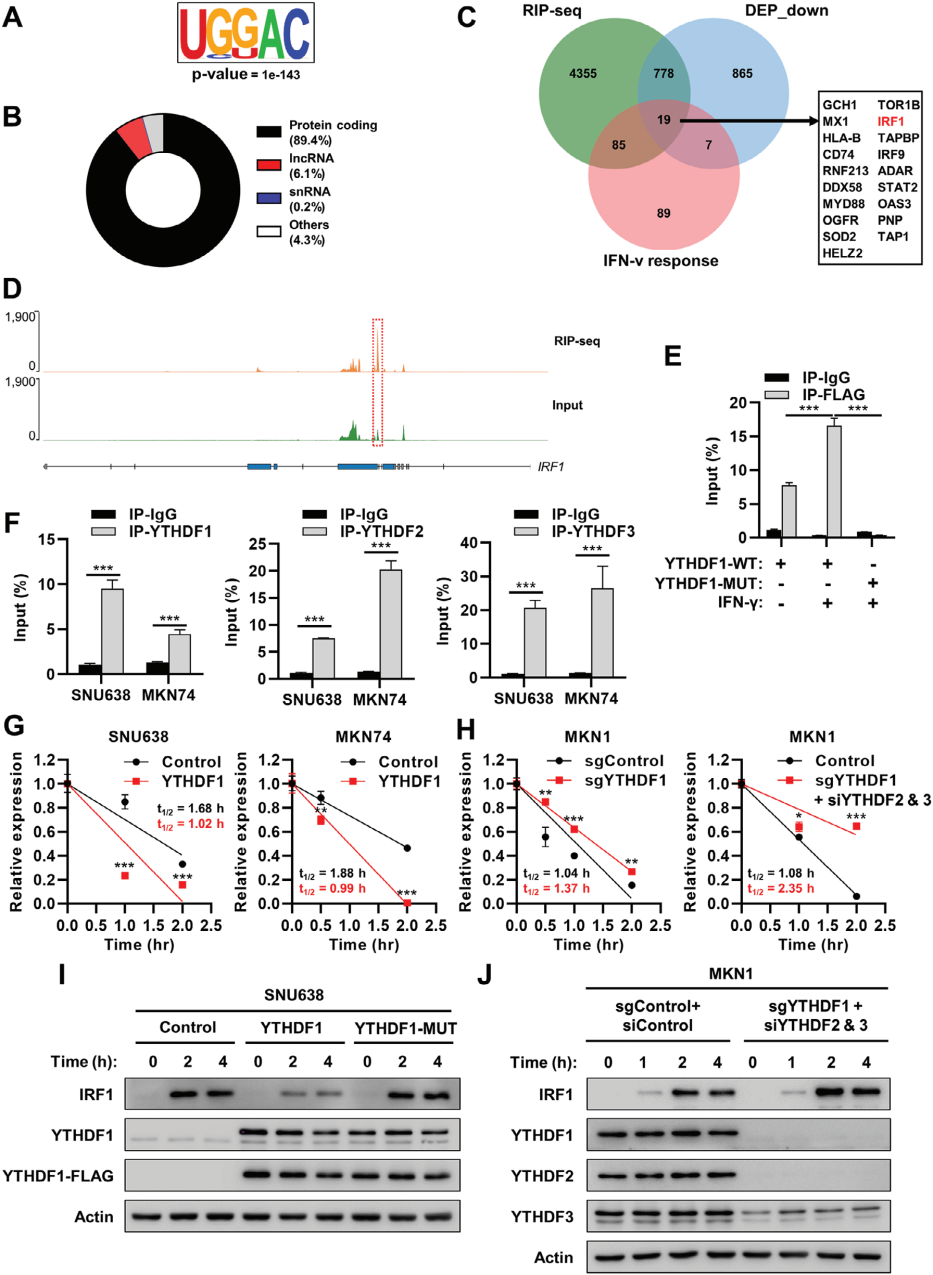
YTHDF1 modulates the IFN‐γ response by regulating IRF1 mRNA stability. A) The consensus binding motif of YTHDF1 in mRNA unveiled through RNA immunoprecipitation sequencing (RIP‐seq). The consensus motif and *p* value of two biological replicates were analyzed using HOMER motif discovery algorithm. B) Pie chart showing the distribution of YTHDF1 binding transcripts in the protein coding, lncRNA, snRNA, and other regions. C) The Venn diagram illustrating the overlap between protein‐coding peaks identified through the union of replicates using RIP‐seq, downregulated differentially expressed proteins (DEPs) under the regulation of YTHDF1, and the “IFN‐γ response” gene set from hallmark gene set. D) Integrative Genomics Viewer showing YTHDF1 RIP‐seq data at the IRF1 transcripts. The y‐axis shows sequencing read counts, and the blue box indicates the predicted YTHDF1 binding sites. E) Binding of YHDF1 to IRF1 mRNA validated by RIP‐qPCR analysis. SNU638 cells were transfected with either FLAG‐tagged YTHDF1 wild‐type (WT) or m6A‐binding defective mutant (YTHDF1‐MUT), and then treated with 10 ng mL^−1^ IFN‐γ for 24 h. Immunoprecipitation was performed using anti‐FLAG antibody. *p* values were calculated using one‐way ANOVA (***p < 0.001). F) Binding of YTHDF1‐3 to IRF1 mRNA validated by RIP‐qPCR analysis. After SNU638 and MKN74 cells were treated with 10 ng mL^−1^ IFN‐γ for 24 h, immunoprecipitation was performed using anti‐YTHDF1, anti‐YTHDF2, or anti‐YTHDF3 antibody. *p* values were calculated using unpaired *t*‐test (***p < 0.001). G) Effect of YTHDF1 overexpression in IRF1 mRNA stability. Relative IRF1 mRNA levels were determined by qPCR after overexpression of YTHDF1in SNU638 (left) and MKN74 cells (right) treated with actinomycin D (10 µg mL^−1^). Serum‐starved cells were treated with 10 ng mL^−1^ IFN‐γ for 24 h, before treatment of actinomycin D. *p* values were calculated using unpaired *t*‐test (**p < 0.01, ***p < 0.001). H) Effect of YTHDF1 or YTHDF1‐3 knockdown in IRF1 mRNA stability. Relative IRF1 mRNA levels were determined by qPCR after YTHDF1 knockdown using Cas9 and single guide RNA (sgRNA) for YTHDF1 (left) or transfection both siRNA targeting YTHDF2 and siRNA targeting YTHDF3 in YTHDF1 stably knocked down MKN1 cells. Serum‐starved cells were treated with 10 ng mL^−1^ IFN‐γ for 24 h, before treatment of actinomycin D (10 µg mL^−1^). *p* values were calculated using unpaired *t*‐test (*p < 0.05, **p < 0.01, ***p < 0.001). I) Effect of YTHDF1 overexpression on the protein levels of IRF1 in the presence of IFN‐γ. Western blot images represent IRF1 protein levels induced by IFN‐γ treatment (1 ng mL^−1^) in YTHDF1‐WT or YTHDF1‐MUT‐overexpressed SNU638 cells. J) Effect of YTHDF1‐3 knockdown on the protein levels of IRF1 in the presence of IFN‐γ. Western blot images represent IRF1 protein levels induced by IFN‐γ (1 ng mL^−1^) in MKN1 cells. Cells were transfected both siRNA targeting YTHDF2 and siRNA targeting YTHDF3 in CRISPR‐based YTHDF1 knockdown (sgControl: control sgRNA; sgYTHDF1: sgRNA for YTHDF1; siControl: control siRNA, sgYTHDF1: single guide RNA for YTHDF1, siYTHDF2: siRNA for YTHDF2, siYTHDF3: siRNA for YTHDF3).

To elucidate the molecular mechanisms by which YTHDFs modulate gene expression in response to IFN‐γ, we performed RNA immunoprecipitation sequencing (RIP‐seq) for overexpressed YTHDF1. Our enriched sequence analysis revealed that YTHDF1 preferentially bound to the DRACH motif (**Figure** **5**A), a consensus sequence motif for m6A modification,^[^ ^17^ ^]^ and protein‐coding genes (Figure 5B). The mRNAs of a total of 5237 genes were enriched in the RIP‐seq data for YTHDF1 (Figure 5C). Among these genes, 19 were downregulated by YTHDF1 overexpression in a proteomic analysis of newly synthesized proteins (Figure 3D) and were categorized as involved in the “IFN‐γ response” in the hallmark gene set (Figure 5C). Of particular interest, we found that IRF1, one of the key proteins that modulate the IFN‐γ response, was a potential target of YTHDF1 (Figure 5D). To verify whether IRF1 is a direct target of YTHDF1, we performed RIP followed by quantitative polymerase chain reaction (RIP–qPCR) and found that wild‐type YTHDF1 could bind to IRF1 transcripts, but the mutant form of YTHDF1 failed to bind, even in the presence of IFN‐γ stimulation (Figure 5E). Moreover, YTHDF2 and YTHDF3 could also bind to the IRF1 transcript, indicating that YTHDFs can regulate IRF1 mRNA (Figure 5F). These interactions were further confirmed with RNA pulldowns using two different IRF1 probes, which revealed specific binding of IRF1 mRNA to YTHDF1‐3 proteins (Figure S7A, Supporting Information).

Next, we investigated the effect of YTHDFs on the mRNA stability of IRF1. In the presence of actinomycin D, overexpression of wild‐type YTHDF1 decreased the half‐life of the IRF1 transcript (Figure 5G). Although knockdown of YTHDF1 slightly increased the half‐life of IRF1 mRNA (Figure 5H), additional downregulation of YTHDF2 and YTHDF3 significantly enhanced the stability of IRF1 mRNA (Figure 5H). In contrast, when assessing newly synthesized mRNAs using 5‐ethynyl uridine (EU) labeling, we observed no difference in EU‐labeled nascent IRF1 mRNA levels between control and YTHDF1 knockdown cells following IFN‐γ treatment (Figure S7B, Supporting Information), indicating that YTHDF1 minimally affects IRF1 mRNA transcription.

When we checked the protein levels of IRF1 in the presence of IFN‐γ, overexpression of wild‐type YTHDF1, but not the m6A‐binding‐defective mutant form of YTHDF1, reduced the protein levels of IRF1 (Figure 5I; Figure S7C, Supporting Information). Although knockdown of YTHDF1 had little effect on the protein levels of IRF1 in the presence of IFN‐γ (Figure S7D, Supporting Information), additional downregulation of YTHDF2 and YTHDF3 significantly increased the IFN‐γ‐induced protein expression of IRF1 in GC cells (Figure 5J; Figure S7E, Supporting Information). Accordingly, knockdown of YTHDFs significantly enhanced the IFN‐γ‐induced expression levels of IRF1 target genes (Figure S7F,G, Supporting Information). These data elucidated the interaction between YTHDFs and IRF1 mRNA, identifying a process that can lead to the degradation of IRF1 mRNA.

### 2.5 Depletion of METTL3 Enhanced the IFN‐γ Response in GC

**Figure 6:**
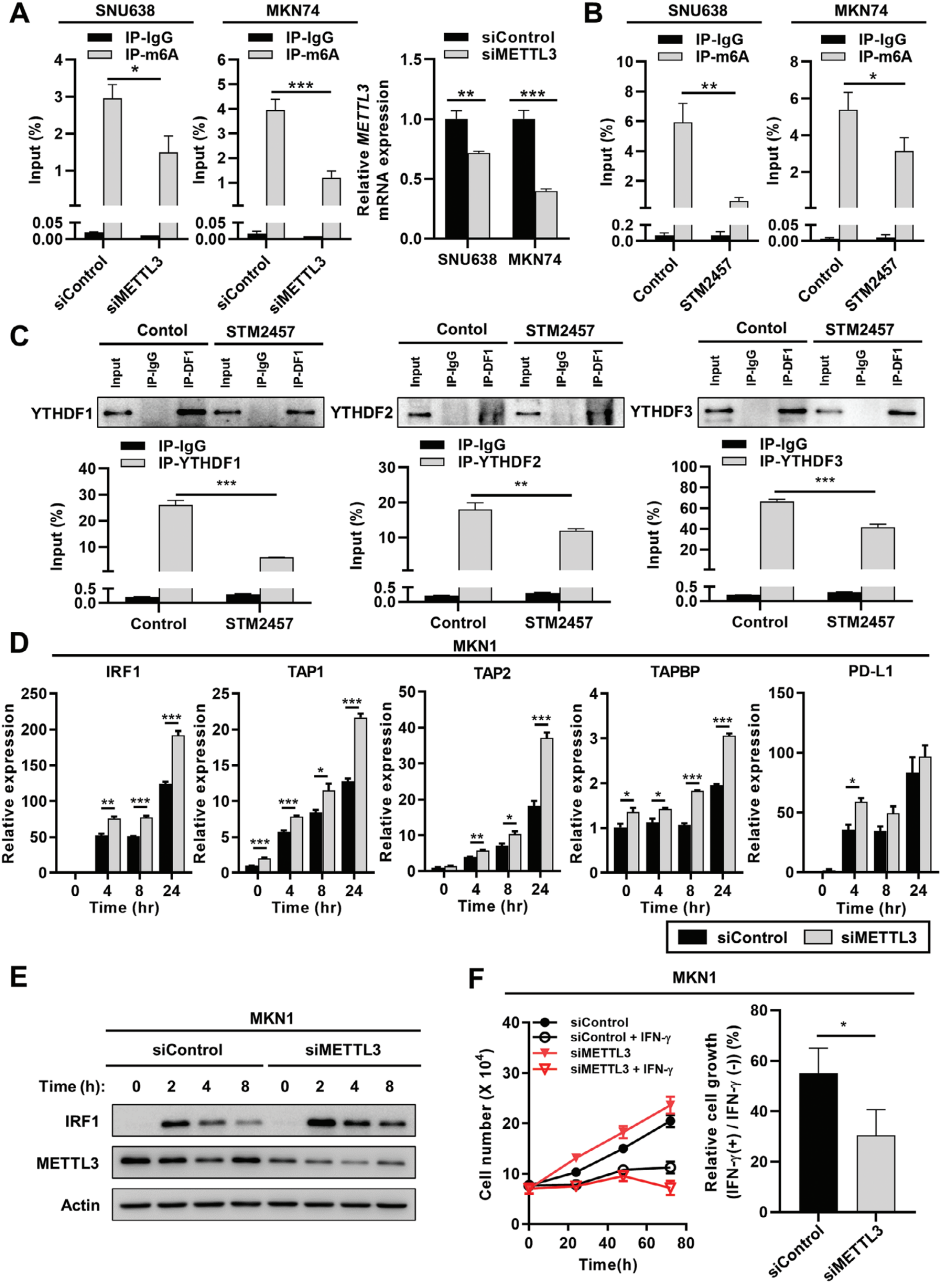
The knockdown of METTL3 enhances the IFN‐γ response in GC cells. A) METTL3 induced m6A modification of IRF1 mRNA validated by MeRIP‐qPCR analysis. SNU638 (left) and MKN74 (right) cells were transfected with siRNA targeting METTL3. After serum starvation for 24 h, cells were treated with 10 ng mL^−1^ IFNγ for 24 h. Immunoprecipitation was performed using anti‐m6A antibody. *p* values were calculated using unpaired *t*‐test (*p < 0.05, ***p < 0.001). B) STM2457 inhibited m6A modification of IRF1 mRNA, as validated by MeRIP‐qPCR analysis. After SNU638 (left) and MKN74 (right) cells were pre‐treated with 10 µm STM2457 for 1 h, cells were treated with 10 ng mL^−1^ IFNγ with 10 µm STM2457 for 24 h. Immunoprecipitation was performed using anti‐m6A antibody. *p* values were calculated using unpaired *t*‐test (**p < 0.01). C) Reduced binding of YTHDF1‐3 to IRF1 mRNA by STM2457 was validated through RIP‐qPCR analysis. After SNU638 cells were pre‐treated with 10 µm STM2457 for 1 h, cells were treated with 10 ng mL^−1^ IFNγ with 10 µm STM2457 for 24 h. Immunoprecipitation was performed using anti‐YTHDF1, anti‐YTHDF2, or anti‐YTHDF3 antibody. *p* values were calculated using unpaired *t*‐test (**p < 0.01, ***p < 0.001). D) Expression of IFNγ‐responsive genes in METTL3 knockdown GC cells. MKN1 cells were transfected with siRNA targeting METTL3. After serum starvation for 24 h, cells were treated with 10 ng mL^−1^ IFNγ, and the mRNA expression levels of IFNγ‐responsive genes were estimated by qPCR relative to the levels of GAPDH (*n* = 3) at indicated time points. Relative values are compared to 0 h in control. *p* values were calculated using unpaired *t*‐test (*p < 0.05, **p < 0.01, ***p < 0.001). E) Effect of METTL3 knockdown on the protein levels of IRF1 in the presence of IFN‐γ. Western blot images represent IRF1 protein levels induced by IFN‐γ (1 ng mL^−1^) in MKN1 cells. Cells were transfected with siRNA targeting METTL3 (siControl: control siRNA, siMETTL3: siRNA for METTL3.). F) Suppression of cell proliferation by IFNγ treatment in METTL3 knockdown GC cells. After serum starvation for 24 h, MKN1 cells were treated with 10 ng mL^−1^ IFNγ for indicated time points and the cell numbers were estimated by trypan blue staining assay (left). Relative cell growth is estimated compared to each IFN‐γ‐untreated control in 72 h (right). *p* values were calculated using unpaired *t*‐test (*p < 0.05).

Next, we investigated whether the regulatory effects of YTHDFs on IRF1 mRNA stability were dependent on RNA m6A modification. After analyzing the RIP‐seq data to predict the m6A motif within IRF1 transcripts, we performed methylated RNA immunoprecipitation (MeRIP)‐qPCR using anti‐m6A antibody and confirmed the anticipated modification of IRF1 transcripts with m6A (**Figure** **6**A,B). To reduce the intracellular m6A levels, we downregulated METTL3, a major component of the m6A methyltransferase complex, using siRNA (Figure 6A) or treated the cells with a highly selective METTL3 catalytic inhibitor, STM2457 (Figure 6B).^[^ ^18^ ^]^ Treatment with either the siRNA for METTL3 or the inhibitor STM2457 resulted in a significant decrease in m6A levels within IRF1 transcripts, as demonstrated by MeRIP–qPCR (Figure 6A,B). Moreover, RIP–qPCR experiments demonstrated that inhibition of METTL3 with STM2457 decreased the binding between YTHDFs and IRF1 transcripts (Figure 6C; Figure S8A, Supporting Information), increasing the mRNA levels of IRF1 (Figure S8B, Supporting Information). Depletion of METTL3 with siRNA enhanced the stability of IRF1 mRNA (Figure S8C, Supporting Information), the IFN‐γ‐mediated mRNA expression levels of IFN‐γ‐regulated genes (Figure 6D; Figure S8D, Supporting Information), the IFN‐γ‐mediated protein expression level of IRF1 (Figure 6E; Figure S8E, Supporting Information), and the inhibition of IFN‐γ‐induced cell proliferation (Figure 6F; Figure S8F, Supporting Information). Altogether, these results support the hypothesis that YTHDFs downregulate IRF1 expression in an m6A‐dependent manner.

### 2.6 YTHDF1 Depletion Enhanced Cancer Immunity in Murine Xenograft Models

To validate the roles of YTHDF1 in immune response downregulation in vivo, we established mouse xenograft models with murine colorectal cancer MC38 cells because mouse syngeneic models for GC were not available. First, we examined the effect of Ythdf1 knockdown on the Ifn‐γ response in MC38 cells using two different sgRNAs. Ythdf1 knockdown had little effect on Stat1 phosphorylation (Figure S9A, Supporting Information) but notably enhanced the expression of Ifn‐γ‐induced genes, including Irf1, in the presence of Ifn‐γ (Figure S9B,C, Supporting Information). Ythdf1 depletion also modestly reduced cell proliferation in vitro, and Ifn‐γ treatment further inhibited cell growth (Figure S9D, Supporting Information). In addition, Ythdf1 overexpression suppressed Ifn‐γ‐mediated expression of Ifn‐γ‐regulated genes, but not in the m6A‐binding defective mutant form of Ythdf1 (Figure S9E, Supporting Information). When we established subcutaneous xenograft models with these cells in immunodeficient non‐obese diabetic severe combined immunodeficiency gamma (NSG) mice, Ythdf1 depletion resulted in a moderate retardation in tumor growth (Figure S10A–C, Supporting Information), suggesting that Ythdf1 depletion impedes the growth of cancer cells, irrespective of host immunity in mouse xenograft models.

**Figure 7:**
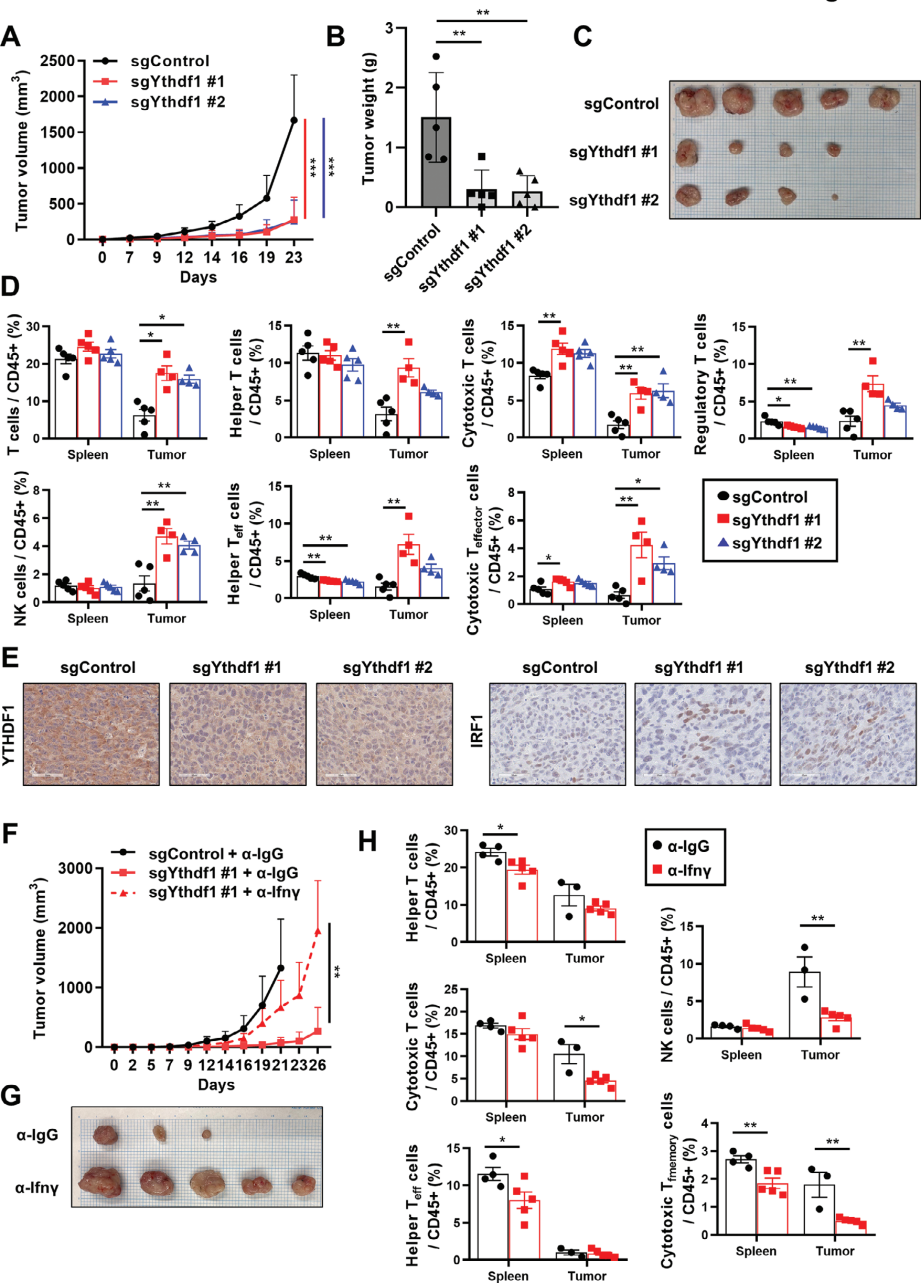
Ythdf1 deficiency enhanced the antitumor response through Ifn‐γ signaling in mouse syngeneic models. A) Tumor growth curves for subcutaneous control and YTHDF1 knockdown MC38 cells in C57BL/6J mice. Tumor volume measurements at indicated time points were calculated using the formula: 0.5 × (length × width^2). *n* = 5 mice per group (sgControl: control sgRNA; sgYthdf1: sgRNA for Ythdf1). *p* values were calculated using one‐way ANOVA (***p < 0.001). B, C) Tumor weight (B) and image (C) of control and YTHDF1 knockdown cell xenograft models (*n* = 5). *p* values were calculated using one‐way ANOVA (**p < 0.01). D) Proportion of T cells and NK cells measured by flow cytometry (21 days after injecting MC38 cells). Flow cytometry analyses were performed to determine the percentage of CD3^+^ T cells, CD3^+^ CD4^+^ CD8^−^ helper T cells, CD3^+^ CD4^−^ CD8^+^ cytotoxic T cells, CD3^+^ CD4^+^ CD8^−^ Foxp3^+^ regulatory T cells, CD3^−^ CD49b^+^ NK1.1^+^ NK cells, CD3^+^ CD4^+^ CD8^−^ CD44^+^ CD62L^−^ effector helper T cells, and CD3^+^ CD4^−^ CD8^+^ CD44^+^ CD62L^−^ effector cytotoxic T cells in spleen and tumor. *p* values were calculated using one‐way ANOVA (*p < 0.05, **p < 0.01). E) Immunohistochemical staining of α‐Ythdf1 (left) and α‐Irf1 (right) were determined. Representative photographs were shown. Scale bars: 50 µm. F) Tumor growth curves for subcutaneous control and YTHDF1 knockdown MC38 cells in C57BL/6J mice treated with α‐IgG or α‐Ifn‐γ. Tumor volume measurements at indicated time points were calculated using the formula: 0.5 × (length × width^2). *n* = 5 mice per group (sgControl: control sgRNA; sgYthdf1: sgRNA for Ythdf1). *p* values were calculated using unpaired *t*‐test (**p < 0.01). G) Image of YTHDF1 knockdown cells treated with α‐IgG or α‐Ifn‐γ in xenograft models (*n* = 5). H) Proportion of T cells and NK cells measured by flow cytometry (26 days after injecting MC38 cells). Flow cytometry analyses were performed to determine the percentage of CD3^+^ T cells, CD3^+^ CD4^+^ CD8^−^ helper T cells, CD3^+^ CD4^−^ CD8^+^ cytotoxic T cells, CD3^−^ CD49b^+^ NK1.1^+^ NK cells, CD3^+^ CD4^+^ CD8^−^ CD44^+^ CD62L^−^ effector helper T cells, and CD3^+^ CD4^−^ CD8^+^ CD44^+^ CD62L^+^ memory cytotoxic T cells in spleen and tumor. *p* values were calculated using unpaired *t*‐test (*p < 0.05, **p < 0.01).

Next, we subcutaneously injected MC38 cells into syngeneic immunocompetent C57BL/6 mice and monitored tumor growth and tumor‐infiltrating lymphocytes (TILs; Figure S11A,B, Supporting Information). Ythdf1 depletion significantly suppressed tumor growth (**Figure** **7**A) and decreased tumor weight and volume (Figure 7B,C) compared with the immunodeficient NSG mouse model (Figure S10A–C, Supporting Information). Accordingly, analyses of TILs demonstrated that Ythdf1 depletion significantly increased the intratumoral infiltration of total and activated immune cells, including helper T cells, cytotoxic T cells, and NK cells (Figure 7D). Immunohistochemical staining confirmed the increased infiltration of CD4^+^ helper T cells and CD8^+^ cytotoxic T cells (Figure S12A–D, Supporting Information). In addition, Ythdf1 depletion significantly increased the intratumoral expression of the Irf1 protein (Figure 7E; Figure S12E, Supporting Information).

To investigate whether the inhibitory effect on tumor growth resulting from Ythdf1 depletion is mediated by Ifn‐γ signaling, we utilized an Ifn‐γ‐blocking antibody in this syngeneic model. The enhanced cancer immunity induced by Ythdf1 depletion was partially due to the enhanced Ifn‐γ signaling because inhibition of Ifn‐γ with blocking antibody increased the tumor growth in Ythdf1 knockdown cells (Figure 7F,G). Treatment with an Ifn‐γ‐blocking antibody significantly suppressed the intratumoral infiltration of cytotoxic T and NK cells (Figure 7H). These data demonstrate the critical role of Ythdf1 in downregulating the immune response in vivo, as evidenced by the enhanced immune cell infiltration and suppression of tumor growth upon Ythdf1 depletion.

To infer the in vivo roles of YTHDF2 and YTHDF3, we first examined the effect of Ythdf2 or Ythdf3 knockdown on Ifn‐γ response in MC38 cells, because in vitro data from MC38 cells have shown strong consistency with animal experiments in Ythdf1 knockdown models (Figure 7A–C; Figure S9C, Supporting Information). The knockdown of Ythdf2 or Ythdf3 enhanced the expression of Ifn‐γ‐induced genes, including Cxcl10, Cxcl9, Tapbp, and Tap1 (Figure S13A,B, Supporting Information). Moreover, in GC cancer tissues from TCGA dataset, analysis of immune cell infiltration based on RNA‐seq data revealed that lower expression levels of YTHDF2 or YTHDF3 were associated with increased infiltration of dendritic cells and CD4^+^ T cells (Figure S13C, Supporting Information). Based on these findings, we established a co‐culture system with human cancer cells and T cells to validate the immune cell activation driven by YTHDF1‐3 inhibition in vitro. Knockdown of YTHDF1‐3 significantly enhanced IFN‐γ‐induced expression levels of cytokines and receptors, including CXCR3, CXCL9, CXCL10, CXCL11, and IL‐6, in the presence of IFN‐γ (Figure S14A, Supporting Information), and these cytokines are known to facilitate T‐cell infiltration and activation.^[^ ^19^, ^20^, ^21^ ^]^ In line with the observed cytokine increase, co‐culturing SNU668 cells with YTHDF1‐3 knockdown significantly enhanced CD69 expression, a T‐cell activation marker, in Jurkat cells following stimulation with anti‐CD3/anti‐CD28 antibodies (Figure S14B, Supporting Information). Furthermore, single cell sequencing data from GC patients revealed an inverse correlation between the expression of YTHDF1 and YTHDF2 and that of IRF1, particularly within tumor cells, T cells, and macrophages (Figure S15A,B, Supporting Information). These findings suggest that YTHDF proteins play a suppressive role in modulating immune responses against cancer in vivo, potentially influencing the tumor microenvironment and immune cell activity.

### 2.7 Increased Human Epidermal Growth Factor Receptor 2 (HER2) Expression is Associated with High YTHDF1 Expression in Patients with GC

**Figure 8:**
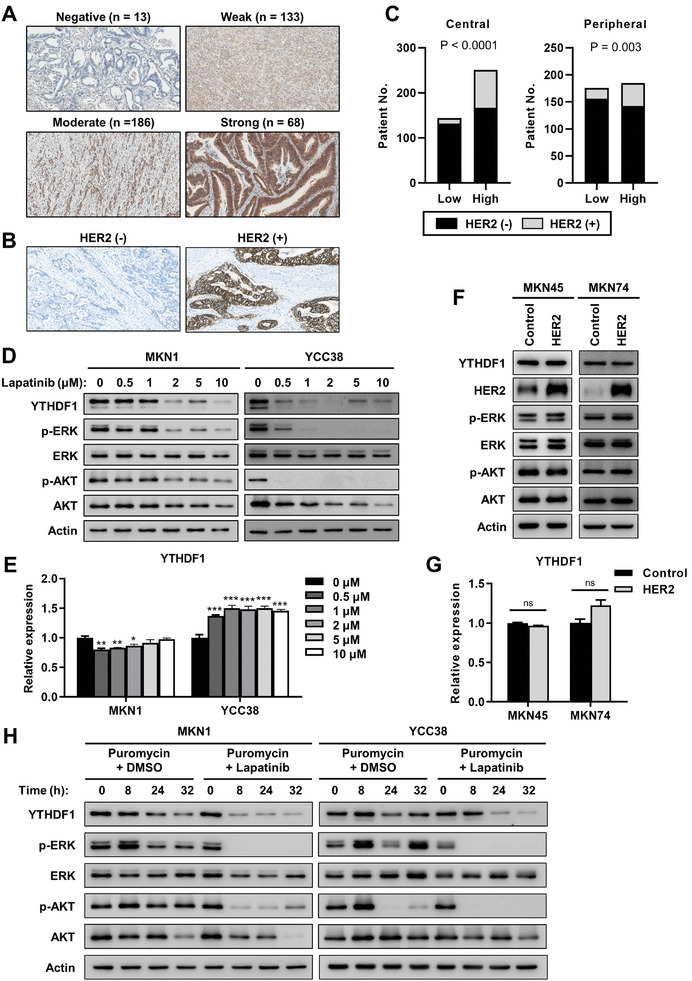
HER2 regulates protein stability of YTHDF1. A,B) Representative images of immunohistochemical (IHC) staining of YTHDF1 (A) and HER2 (B) in 400 gastric cancer tissues. C) HER2 positive rate in samples with low YTHDF1 expression (*n* = 146) and high YTHDF1 expression samples (*n* = 254) in central region of gastric cancer tissue (left). HER2 positive rate in samples with low YTHDF1 expression (*n* = 197) and high YTHDF1 expression samples (*n* = 192) in peripheral region of gastric cancer tissue (right). *p* values were calculated using χ^2^ test. D) Protein levels of YTHDF1 after treatment of lapatinib in MKN1 (left) and YCC38 cells (right). Protein levels were evaluated by western blotting. E) Relative mRNA levels of YTHDF1 after treatment of lapatinib in MKN1 (left) and YCC38 cells (right). The mRNA levels were determined by qPCR relative to the levels of GAPDH. Relative values are estimated compared to each 0 µm sample. *p* values were calculated using one‐way ANOVA (*p < 0.05, **p < 0.01, ***p < 0.001). F) Protein levels of YTHDF1 after transfected HER2 plasmid in MKN45 (left) and MKN74 cells (right). Protein levels were evaluated by western blotting. G) Relative mRNA levels of YTHDF1 after transfected HER2 plasmid in MKN45 (left) and MKN74 cells (right). The mRNA levels were determined by qPCR relative to the levels of GAPDH. Relative values are estimated compared to control sample. *p* values were calculated using unpaired *t*‐test. H) Effect of HER2 inhibition in YTHDF1 protein stability. Relative YTHDF1 protein levels were evaluated by western blotting after treatment of puromycin (2 µg mL^−1^) and lapatinib (10 µm) in MKN1 (left) and YCC38 cells (right).

To elucidate the role of YTHDF1 in patients with GC, we analyzed YTHDF1 protein expression in a second cohort of in‐house tissues from patients with GC (*n* = 400) using immunohistochemistry.^[^ ^22^ ^]^ In central tumor regions, 63.5% of patients showed high expression of YTHDF1 (moderate, *n* = 186; strong, *n* = 68), whereas 36.5% of patients showed low expression of YTHDF1 (negative, *n* = 13, weak, *n* = 133) (**Figure** **8**A; Table S2, Supporting Information). In the peripheral invasive margin of tumors, 49.4% of patients showed high expression of YTHDF1 (moderate, *n* = 170; strong, *n* = 22), whereas 50.6% of patients showed low expression of YTHDF1 (negative, *n* = 28, weak, *n* = 169) (Table S3, Supporting Information). When we analyzed the associations between YTHDF1 expression and clinical parameters, high expression of YTHDF1 was associated with female sex, older age, and lymphatic metastasis in both central and peripheral tumor regions (Tables S2 and S3, Supporting Information).

Because there are more patients with GC with increased YTHDF1 gene expression than patients with increased gene copy number, alternative mechanisms beyond copy number amplification may contribute to increased YTHDF1 gene expression. When we analyzed the associations between YTHDF1 expression and clinical parameters, HER2 expression was highly correlated with YTHDF1 expression (Figure 8B,C). When we treated HER2‐positive GC cell lines (MKN1 and YCC38) with the HER2 inhibitor lapatinib, the protein levels of YTHDF1 were significantly reduced, concomitant with decreases in extracellular signal‐regulated kinase (ERK) and protein kinase B (AKT) phosphorylation (Figure 8D). However, following lapatinib treatment, YTHDF1 mRNA expression either changed minimally or increased (Figure 8E). Yet, HER2 overexpression in HER2 low‐expressing GC cell lines had limited effects on YTHDF1 protein and mRNA expression levels, probably due to the absence of additional activation of the HER2 downstream signaling pathway, as indicated by minimal changes in ERK and AKT phosphorylation (Figure 8F,G). When cells were treated with the translation inhibitor puromycin, the protein half‐life of YTHDF1 was significantly reduced in lapatinib‐treated cells (Figure 8H). These data indicate that heightened HER2 expression positively regulates YTHDF1 expression at the protein stability level.

## 3 Discussion

Cancer immunity is shaped by tumor‐intrinsic and microenvironment‐related factors, which are regulated through multiple layers of gene expression control. RNA m6A modification plays a crucial role in the regulation of post‐transcriptional gene expression, and previous studies have emphasized the significant impact of RNA m6A modifications on cancer immunity.^[^ ^23^, ^24^, ^25^ ^]^ In addition, a lower level of m6A modification is associated with increased immune infiltration and enhanced response to immunotherapy.^[^ ^25^ ^]^ This study reveals the inhibitory roles of YTHDF paralogs, the m6A reader proteins, in cancer immunity related to GC by modulating the mRNA stability of IRF1, a key regulator of the IFN‐γ response. These findings propose a novel molecular mechanism for the regulation of cancer immunity through RNA m6A modification‐mediated post‐transcriptional regulation.

YTHDF1 has been reported to be upregulated in multiple cancer types, thereby promoting cancer growth.^[^ ^26^, ^27^, ^28^ ^]^ Copy number amplification of YTHDF1 genes was identified as a genetic cause underlying the increased expression of YTHDF1 in tumor tissues, as demonstrated in both our in‐house GC and the TCGA GC cohorts. In these cohorts, amplification or gain of the YTHDF1 gene was detected in a substantial proportion of GC tumors (Figure 1A). In addition, HER2 expression was highly associated with YTHDF1 upregulation at the protein level (Figure 8). HER2 overexpression is detected in ≈7.3–20.2% of patients with advanced gastric/gastroesophageal junction adenocarcinoma, which led to the emergence of trastuzumab, an anti‐HER2 antibody, as the established first‐line treatment for patients with advanced metastatic HER2‐positive GC.^[^ ^29^ ^]^ Considering a previous study indicating that ERK1/2‐mediated phosphorylation enhances YTHDF2 stability,^[^ ^30^ ^]^ along with the high sequence similarity between YTHDF1 and YTHDF2, it is probable that HER2 activation may enhance YTHDF1 protein stability through a phosphorylation‐dependent mechanism. However, this hypothesis requires experimental validation. Taken together, our findings indicate that augmented HER2 signaling contributes to the upregulation of YTHDF1 expression, further implying a critical role for YTHDF1 in sustaining the activation of downstream signaling pathways associated with HER2‐positive cancers.^[^ ^31^ ^]^ Furthermore, YTHDF1 upregulation is implicated in immune evasion mechanisms observed in HER2‐positive GC.^[^ ^32^ ^]^

IFN‐γ has been reported to be associated with cancer immunity, and enhancing its reactivity is crucial for overcoming resistance to immunotherapy.^[^ ^33^ ^]^ An impaired IFN‐γ response leads to diminished tumor antigen presentation and reduced PD‐L1 expression and subsequently contributes to primary resistance against PD‐1 blockade therapies.^[^ ^34^, ^35^ ^]^ Upon investigating the mRNA targets of YTHDF1 based on proteomics and RIP‐seq data, we discovered that YTHDF1 directly binds to the m6A‐modified site within the protein‐coding region of IRF1 mRNA, consequently triggering its degradation. Given that IRF1 serves as a master regulator of IFN‐γ responsiveness, governing diverse IFN‐γ signaling pathways, including those involved in anti‐cancer apoptotic pathways^[^ ^36^ ^]^ and enhanced immunogenicity,^[^ ^37^ ^]^ the regulation of IFN‐γ signaling serves as a fundamental molecular mechanism underlying the immune evasion effect of YTHDF1. These results are supported by our finding that blocking IFN‐γ effectively abolishes the immune‐enhancing effect observed upon YTHDF1 depletion (Figure 7F,G).

YTHDF1 has emerged as a critical factor in both tumor‐intrinsic and microenvironment‐related processes. Recent studies have highlighted the role of tumor‐intrinsic YTHDF1 in regulating cell proliferation and drug resistance, particularly through the modulation of frizzled class receptor 7 (FZD7),^[^ ^38^ ^]^ ubiquitin‐specific protease 14 (USP14),^[^ ^39^ ^]^ and poly(ADP‐ribose) polymerase 1 (PARP1).^[^ ^40^ ^]^ Furthermore, YTHDF1 has been implicated in the regulation of the tumor immune microenvironment, particularly in immune evasion, by modulating the expression of major histocompatibility complex class I (MHC‐I),^[^ ^41^ ^]^ the proto‐oncogene NFĸB subunit RELA,^[^ ^42^ ^]^ and PD‐L1/V domain Ig suppressor of T‐cell activation (VISTA).^[^ ^43^ ^]^ Our findings further suggest that elevated YTHDF1 expression is associated with the downregulation of immune‐related gene sets and the reduced abundance of CD8^+^ T cells and NK cells (Figure 1E,F). In addition to the dysregulation of genes and proteins related to cancer immunity, YTHDF1 overexpression led to the enhanced synthesis of proteins associated with cell cycle progression and DNA synthesis, as revealed by proteomic analysis (Figure S3E, Supporting Information). These findings underscore the potential of targeting YTHDF1 as a promising approach in cancer therapeutics, as it is capable of modulating both tumor cell‐intrinsic factors and immune‐related aspects of the tumor microenvironment.

Diverging perspectives exist regarding the precise molecular functions of YTHDF paralogs. Although YTHDF2 is primarily associated with mRNA degradation, YTHDF1 and YTHDF3 have been proposed to play roles in mRNA translation processes.^[^ ^11^ ^]^ Especially for the function of YTHDF1, some studies have shown that YTHDF1 may contribute to cellular metabolism regulation by enhancing the stability of specific mRNAs, such as MYC and HK2.^[^ ^44^, ^45^ ^]^ In addition, YTHDF1 overexpression enhanced the immunosuppressive functions of IFN‐γ‐licensing mesenchymal stem cells by increasing the mRNA stability of immunosuppressive molecules, including IDO1, PD‐L1, ICAM1, and VCAM1.^[^ ^46^ ^]^ However, data elucidating the mechanisms by which YTHDF1 stabilizes mRNAs remain limited. Conversely, other studies suggest that YTHDF1 may promote mRNA degradation, as all three YTHDF proteins (YTHDF1‐3) share highly similar domain structures and interact with the CCR4‐NOT RNA degradation complex.^[^ ^47^, ^48^ ^]^ Our data suggest that YTHDF1‐3 exhibit similar effects in gastric cancer, particularly within interferon gamma signaling, as demonstrated by gene set enrichment analysis (Figure S5, Supporting Information). YTHDF1 overexpression was associated with reduced IRF1 mRNA stability (Figure 5G) and decreased protein translation (Figure 5C), whereas YTHDF1 knockdown significantly enhanced IRF1 mRNA stability (Figure 5H). Concomitant depletion of YTHDF2 or YTHDF3, alongside YTHDF1 knockdown, resulted in further augmentation of IFN‐γ‐induced gene expression and IFN‐γ‐mediated suppression of cell proliferation (Figure 2). Moreover, YTHDF1–3 exhibited direct binding toward IRF1 mRNA (Figure 5F; Figure S7A, Supporting Information), and simultaneous depletion of YTHDF1–3 led to increases in IRF1 mRNA stability and protein expression (Figure 5H,J). A plausible explanation for this discrepancy is that YTHDF1 functions in a target‐dependent manner, influenced by specific m6A stoichiometry, mRNA sequences, and neighboring RNA‐binding proteins, which may alter the binding partners of YTHDF1. However, further evaluation is required to determine the precise molecular functions of each YTHDF protein, as a recent study suggested that the triple knockdown of YTHDF1–3 promotes global mRNA stabilization by increasing P‐body formation.^[^ ^49^ ^]^

METTL3 functions as the catalytic core in the methyltransferase complex,^[^ ^50^ ^]^ and there is growing interest in small‐molecule inhibitors targeting METTL3 for potential cancer treatments.^[^ ^18^ ^]^ By analyzing RIP‐seq data, we predicted the m6A motif in the IRF1 transcript, which was verified through MeRIP–qPCR (Figure 6A,B). Knockdown of METTL3 in GC substantially reduced m6A modifications in IRF1 transcripts in the presence of IFN‐γ, implicating METTL3 in the direct regulation of m6A within IRF1 mRNAs. Comprehensive exploration of METTL3 depletion revealed a significant increase in IFN‐γ‐mediated expression of IFN‐γ‐regulated genes and a decrease in cell proliferation (Figure 6D–F; Figure S8C–F, Supporting Information). The association of m6A modification with reduced levels of IRF1 mRNA, combined with the role of YTHDFs as m6A readers that mediate mRNA degradation, strongly suggests that YTHDF proteins primarily mediate the m6A‐dependent regulation of IRF1 mRNA. Taken together, the enhanced m6A modification of IRF1 mRNA facilitated by METTL3 led to the recruitment of YTHDFs, resulting in the suppression of IRF1 expression and subsequently diminishing the response of IFN‐γ.

A previous study reported that the m6A methyltransferase Mettl3/14 complex regulates the Ifn‐γ–Stat1–Irf1 axis in mouse colorectal cancer models.^[^ ^51^ ^]^ The proposed molecular mechanism involves the degradation of *Stat1* and *Irf1* mRNAs via *Ythdf2*, resulting in enhanced cancer immunity through the inhibition of RNA m6A modifications and the effect of *Ythdf2*. Compared with the previous report, our findings revealed that YTHDF‐mediated regulation of IFN‐γ signaling primarily occurred through the modulation of IRF1 mRNA stability, independent of STAT1. Furthermore, we observed that the stability of IRF1 mRNA was regulated by all three YTHDF proteins. Despite some differences, our data validated the YTHDF‐mediated regulation of IRF1 and IFN‐γ signaling in human cancer cells, affirming its relevance across species.

## 4 Conclusion

Our findings demonstrated that YTHDF1 was upregulated in GC, which correlated with DNA copy number amplification of the YTHDF1 gene and high HER2 expression. High YTHDF1 expression is associated with poorer overall survival and reduced cancer immunity, particularly affecting the IFN‐γ response of cancer cells. YTHDF2 and YTHDF3, along with YTHDF1, impede the response to IFN‐γ by degrading IRF1 mRNA, and YTHDF2 and YTHDF3 exhibit compensatory effects in the absence of YTHDF1. Furthermore, depletion of Ythdf1 leads to decreased tumor growth and enhanced infiltration of immune cells in a mouse syngeneic tumor model. These findings unveil novel molecular mechanisms underlying the regulation of cancer immunity and IFN‐γ signaling, offering insights into a promising therapeutic strategy to enhance the effectiveness of immune checkpoint therapy.

## 5 Experimental Section

### Cell Culture

The human gastric cancer cell lines (MKN1, MKN74, SNU638, SNU668, and YCC38), mouse colorectal cancer cell line (MC38), and 293FT cells were obtained from Korean Cell Line Bank. MKN1, MKN45, MKN74, SNU638, and SNU668 cells were cultured in RPMI 1640 (Life Technologies), YCC38 cells were cultured in MEM (Life Technologies), and MC38 and 293FT were cultured in DMEM (Life Technologies). All the medium were supplemented with 10% FBS (Life Technologies) and 1% penicillin streptomycin (Life Technologies). All cells were incubated at 37 °C in 5% CO_2_.

### Cell Transfection, Lentiviral Infection, and IFN‐γ Treatment

Transfection of siRNA was performed using lipofectamine 2000 (Invitrogen) and transfection of plasmid was performed using LT‐1 (Takara) for 48 h. For lentiviral single guide RNA (sgRNA) production, sgRNA was constructed with lentiCRISPR v2 vector. Lentiviral vector, VSVg, and psPAX2 were cotransfected in 293FT cells with lipofectamine 2000 for 72 h. Viral particles were concentrated using Lenti‐X concentrator (Takara) and used to infect cells with 8 µg mL^−1^ polybrene for 24 h. After infection, cells were selected in puromycin containing medium for 72 h. The sgRNA sequences targeting the human YTHDF1 gene for knockdown were 5′‐TGCTCACTACGAGAAGCGCC‐3′ or 5′‐AAATGGTTCGTTACATCAGA‐3′ and those targeting the mouse Ythdf1 gene were 5′‐AGCAGCCACTTCAACCCCGC‐3′ or 5′‐TGAACACGGCAACAAGCGCC‐3′. Cells were serum‐starved for 24 h prior to IFN‐γ treatment.

### Array Comparative Genomic Hybridization (aCGH)

Gastric cancer patient sample collection and aCGH were performed as previously described.^[^ ^13^ ^]^ Briefly, total DNA was extracted from gastric cancer samples and paired normal gastric tissue samples. A custom‐designed 1 million copy number variation (CNV) genotyping array was employed for chromosomal analysis. Following aCGH experiments, images were analyzed using Feature Extraction Software 10.5.1.1 (Agilent Technologies) and calculated with ADM2 statistical algorithm.

### Cell Type Proportion Analysis

The relationship between immune cell infiltration and YTHDF1 mRNA expression was investigated in ten samples with the highest and lowest YTHDF1 expression in the TCGA GC cohort. The proportion of CD8^+^ T cells, activated NK cells, and M0 macrophages were quantified with LM22 (22 immune cell types) matrix file using CIBERSORT (https://cibersortx.stanford.edu/).

### Quantative Real‐Time PCR (qPCR)

Total RNA from cells was isolated using RNeasy Plus Mini Kit (Qiagen) according to the manufacturer's instruction. One microgram of RNA was reverse transcribed to make cDNA using Maxime RT PreMix (Intron Biotechnology) and quantitative real‐time PCR was performed using SYBR Green Master Mix (Bio‐rad) with Bio‐rad CFX96. GAPDH mRNA was used as an internal control. The used primers are listed in Table S1 (Supporting Information).

### RNA Stability Assay

Gastric cancer cells were seeded in 12‐well plate at 50% confluency. After seeding, cells were preincubated in serum‐free medium for 24 h and treated with 10 ng mL^−1^ IFN‐γ for 24 h. After stimulation, cells were treated with 10 µg mL^−1^ actinomycin D in serum free medium. Total RNA was extracted and quantified by qPCR.

### Western Blot Analysis

Cells were lysed on ice for 30 min using RIPA buffer (Thermo Scientific) with a protease inhibitor cocktail (Roche) and a phosphatase inhibitor cocktail (Roche). Lysate was centrifuged at 13 200 rpm at 4 °C for 15 min. Then, supernatant was collected and determined the protein concentration by BCA assay (Thermo Scientific). Equal amounts of proteins were fractionated by SDS‐PAGE and transferred to nitrocellulose membranes. Membranes were blocked in 5% skim milk in TBS with tween 20 for 1 h and blotted with primary antibodies overnight at 4 °C. After five times washing, membranes were incubated with secondary antibodies for 1 h at RT. Membrane images were captured using the AI680 (GE Healthcare).

### Protein Stability Assay

Gastric cancer cells were seeded in six‐well plate at 50% confluency. After seeding, cells were cotreated with 10 µm lapatinib and 2 µg mL^−1^ puromycin in medium. Total protein was extracted and evaluated by western blot analysis.

### Cell Proliferation Assay

Cells were harvested using trypsin‐EDTA (Life Technologies) and stained with trypan blue dye (Merck Millipore). The number of live cells were counted using a LUNA automated cell counter (Logos Biosystems).

### Proteomic Analysis for Newly Synthesized Proteins

Cells were starved in RPMI without methionine for 1 h, cotreated 50 µm L‐azidohomoalanine (AHA) and 10 ng mL^−1^ IFN‐γ in methionine free medium for 4 h and harvested. Newly synthesized proteins were isolated using the Click‐&‐GO protein enrichment kit (Click Chemistry Tools) according to the manufacturer's instruction. In brief, cell pellets were lysed using lysis buffer containing a protease inhibitor by sonication on ice. The click reaction was performed with the lysate and alkyne agarose resin overnight. For reduction and alkylation of resin bound proteins, the resin was incubated with 10 mm DTT and 40 mm iodoacetamide solution and proteins were digested by trypsin. Proteomic data were collected by liquid chromatography and mass spectrometry and processed using proteome discoverer 2.2 (Thermo Fisher).

### RNA Immunoprecipitation (RIP)‐Sequencing Analysis and RIP‐qPCR

RNA was isolated using the Magna RIP RNA‐Binding Protein Immunoprecipitation kit (Merck Millipore) according to the manufacturer's instruction. In brief, cells were resuspended in RIP Lysis Buffer and immunoprecipitated with antibody to FLAG or IgG with protein A/G magnetic beads overnight at 4 °C. After six times washing, immunoprecipitated fractions were digested with proteinase K buffer. Then, RNA was extracted by phenol‐chloroform extraction method. Input RNA was also extracted by same method. For RNA sequencing, cDNA library was generated using SMARTer Stranded RNA library kit (Takara) and sequenced using Illumina NovaSeq platform. For qPCR, total RNA was reverse transcribed to cDNA using Maxime RT PreMix (Intron Biotechnology) and qPCR was performed using SYBR Green Master Mix (Bio‐rad) with Bio‐rad CFX96. Primer sequences to amplify IRF1 mRNA for RIP‐qPCR were 5′‐TTCGCTGTGCCATGAACT‐3′ and 5′‐GGAAGCATCCGGTACACTC‐3′.

### RNA Pull‐Down Assay

Cells were UV‐crosslinked at 4000 J m^−^^2^ using a 254 nm wavelength in 15 cm plates with cold DPBS and subsequently harvested. The cell pellets were lysed on ice in immunoprecipitation (IP) lysis buffer (Thermo Scientific) supplemented with a protease inhibitor cocktail (Roche) and a RNase inhibitor (Promega). Following lysis, the lysate was centrifuged at 13 000 × g for 20 min at 4 °C, and the supernatant was transferred to streptavidin magnetic beads (Thermo Scientific) conjugated with a 5′ biotin‐labeled DNA probe or a poly(A)_25_ probe (20 mm Tris‐Cl (pH 7.5), 50 mm NaCl, 2 mm MgCl_2_ and 0.1% Tween‐20) for 1 h at room temperature. After washing the beads six times with washing buffer (20 mm Tris (pH 7.5), 10 mm NaCl and 0.1% Tween‐20), the beads were incubated in sample buffer for 5 min at 100 °C. The supernatants were then analyzed by Western blot. DNA probe sequences to targeting IRF1 mRNA were 5′‐GTAGACTCAGCCCAATATCCC‐3′ and 5′‐CTCATCTGTTGTAGCTTCAGAG‐3′.

### Nascent RNA qPCR

Cells were cotreated with 0.5 mm 5‐ethynyl uridine (EU) and 10 ng mL^−1^ IFN‐γ in serum‐free medium for 2 h, after which RNA was extracted using the phenol‐chloroform extraction method. Nascent RNA was isolated using the Click‐iT Nascent RNA Capture Kit (Thermo Scientific) following the manufacturer's instructions. Briefly, a click reaction was performed between EU‐labeled RNA and azide‐biotin, followed by purification using Streptavidin T1 magnetic beads. The isolated nascent RNA was then reverse‐transcribed into complementary DNA (cDNA) using the SuperScript VILO cDNA synthesis kit (Thermo Scientific). Quantitative qPCR was conducted using SYBR Green Master Mix (Bio‐Rad) with the Bio‐Rad CFX96. Primer sequences to amplify nascent IRF1 RNA for qPCR were 5′‐TTCGCTGTGCCATGAACT‐3′ and 5′‐GGAAGCATCCGGTACACTC‐3′.

### MeRIP‐qPCR

The fragmentation of 10 µg of total RNA was conducted using RNA fragmentation reagent (Thermo Fisher Scientific) at 94 °C for 3 min, followed by the immediate addition of 0.5 m EDTA. Fragmented RNA was cleaned up with Oligo Clean & Concentration kit (Zymo research). Protein A/G beads (Thermo Fisher Scientific) conjugated with anti‐m6A (Synaptic System) or anti‐IgG (Santa Cruz biotechnology, Inc.) in IP buffer (1 m NaCl, 200 mm Tris‐HCl (pH 7.4) and 1% IGEPAL CA‐630) for 30 min at room temperature. After three times beads washing with IP buffer, beads were incubated with fragmented RNA in MeRIP buffer (1 m NaCl, 200 mm Tris‐HCl (pH 7.4) and 1% IGEPAL CA‐630, and 40 U µL^−1^ RNasin Ribonuclease Inhibitor (Promega)). After immunoprecipitation, beads were washed five times with IP buffer and eluted by phenol‐chloroform extraction method. Purified m6A modified RNA was reverse transcribed to make cDNA using Maxime RT PreMix (Intron Biotechnology) and quantitative qPCR was performed using SYBR Green Master Mix (Bio‐rad) with Bio‐rad CFX96. Primer sequences to amplify IRF1 mRNA for MeRIP‐qPCR were 5′‐TTCGCTGTGCCATGAACT‐3′ and 5′‐GGAAGCATCCGGTACACTC‐3′.

### Animal Experiments

C57BL/6J mice and NSG mice (4 weeks old, female) were obtained from the Orient Bio (Seongnam, South Korea) and adapted for 1 week. Mice received appropriate care in accordance with the guidelines set forth by the Institutional Animal Care and Use Committee (IACUC) of Seoul National University (SNU‐210422‐4‐4). To generate mouse syngeneic tumor model, mouse colorectal cancer MC38 cells (2 × 10^5^) were suspended in 0.1 mL OPTI‐MEM (Life Technologies) and subcutaneously injected into mice. Tumor volume was measured every 2–3 days and calculated by 0.5 × (length × width^2^). After 2‐3 weeks, tumor‐bearing mice were sacrificed and measured for tumor weight. To block IFN‐γ signaling, mice bearing sgControl or sgYthdf1 MC38 cells were intraperitoneally injected with 250 µg of anti‐mouse IFN‐γ antibody (BioXCell) or isotype control antibody one day prior to tumor challenge. Following this initial treatment, the same dosage was administered every 2–3 days thereafter.

### Tumor‐Infiltrating Lymphocyte (TIL) Analysis

Fresh dissected tumors were digested with collagenase D (15 mg mL^−1^) and DNase I (1 mg mL^−1^) in RPMI medium containing 5% FBS for 1 h at 37 °C. After incubation, tumors were passed through 40 µm cell strainer. To obtain TIL, tumor cells were resuspended with 30% percoll, loaded onto 70% percoll and centrifuged. Then, the lymphocytes in middle layer were collected and RBCs were lysed using ACK lysing buffer (Life Technologies). Spleen was dissected, filtered by 40 µm cell strainer, and resuspended with ACK lysing buffer.

### Flow Cytometric Analysis

Lymphocytes were blocked with Fc blockade (Invitrogen) for 15 min and incubated with primary antibody in dark for 30 min. After samples were washed with FACS buffer and detected by BD LSRFortessa. For detecting regulatory T cells, mouse regulatory T cell staining kit (Life Technologies) was used. Data were analyzed by the FlowJo program.

### Immunohistochemistry

Mouse tumor tissues were fixed in 4% paraformaldehyde for 48 h at 4 °C and embedded in paraffin. Paraffin‐embedded tissue sections were stained with hematoxylin and immunohistochemically stained with antibodies against Ythdf1, Irf1, Cd4, and Cd8 according to the manufacturer's instruction with Bond‐RXm (Leica biosystems). Stained samples were scanned using an Aperio AT2 (Leica biosystems). Ten areas with the same size were randomly selected and analyzed using a QuPath‐0.4.4.

To estimate the human epidermal growth factor receptor 2 (HER2) status in GC patient cancer tissues, a total of 400 consecutive patients diagnosed with stage II and III GC who underwent surgical resection at Seoul National University Bundang Hospital between 2006 and 2013 were enrolled an increase from the number previously described.^[^ ^22^ ^]^ This study was approved by the institutional review board of the Seoul National University Bundang Hospital (No. B‐2401‐879‐103), in accordance with the Declaration of Helsinki. All patients received fluoropyrimidine‐based (5‐FU) adjuvant chemotherapy after surgery. Retrospective collection of clinicopathological characteristics was performed through examination of medical records and pathology reports. 2‐µm formalin‐fixed and paraffin‐embedded tissue sections were constructed and were selected from both the center of the tumor (CT) and invasive margin (IM). Immunohistochemistry for HER2 was performed human GC tissue microarray (TMA) sections using anti‐HER2 antibody (Ventana Medical Systems). HER2 IHC score was evaluated according to the GC guideline.^[^ ^52^ ^]^ HER2 staining was assessed and scored as follows: HER2‐negative for tumors that scored IHC negative, HER2‐positive for tumors that scored IHC 1+, IHC 2+ or IHC 3+ in either the CT or IM areas.

### Co‐Culture Assay

Gastric cancer cells were seeded in a 12‐well plate at 50% confluency. Following seeding, the cells were preincubated in serum‐free medium for 24 h and subsequently treated with 10 ng mL^−1^ IFN‐γ for 24 h. Jurkat cells were then added directly to the tumor cells and stimulated with soluble anti‐CD3 (1 µg mL^−1^), anti‐CD28 (0.5 µg mL^−1^), and anti‐mouse IgG (2.3 µg mL^−1^) antibodies for 8 h. After incubation, Jurkat cells were harvested for flow cytometry analysis.

### Single Cell RNA Sequencing Analysis

Publicly available single‐cell RNA sequencing data from six patients, comprising ten samples (three primary tumors, six metastatic tumors, and one adjacent non‐tumor tissue sample) were used.^[^ ^53^ ^]^ To address zero‐count sparsity in the single‐cell dataset, the MAGIC algorithm was applied to impute missing values in log‐normalized expression counts, enhancing the detection of correlation signals.^[^ ^54^ ^]^ Spearman correlation coefficients were calculated for YTHDF proteins (YTHDF1, YTHDF2 and YTHDF3) with IRF1 in tumor tissue, as well as across different cell types (T cell, B cell, dendritic cell and macrophage) to capture cell‐type‐specific trends. Correlation significance was assessed with Spearman r values and *p* values, with *p* < 0.05 considered significant.

### Data Availability

RNA‐seq data of human gastric cancer cell line SNU638 treated with IFN‐γ had been deposited to the gene expression omnibus (GEO) GSE270417 and RIP‐seq data for YTHDF1 treated with IFN‐γ in SNU638 had been deposited in the GEO GSE270604.

### Statistical Analysis

Graphs were generated and statistical analysis was performed using Prism (GraphPad). Statistical details of experiments, including number of experiments, statistical test, and statistical significance (*p* value) were reported in the figure legends. Independent experiments were performed to define the reproducibility of the results. Statistical analyses were performed using SPSS 22.0 software (SPSS Inc., Chicago, IL). GraphPad Prism 8.0 (GraphPad Software, San Diego, CA) was applied to plot the data. Student's *t*‐test and one‐way analysis of variance (ANOVA) were used to assess the significance of differences. *p* < 0.05 was considered statistically significance (*p < 0.05, **p < 0.01, and ***p < 0.001). All values were expressed as the means ± standard deviation.

## Conflict of Interest

The authors declare no conflict of interest.

## Author Contributions

D.J., J.‐Y.A., and S.‐Y.C. designed the research. J.O., S.S., S.‐J.L., J.K., S.E.L., Y.Y., D.K., S.L., H.R.J., Y.O., K.K., and H.S.L. performed the experiments. D.J., C.H., S.K., K.K., H.S.L., J.‐Y.A., and S.‐Y.C. analyzed data. D.J., J.‐Y.A., and S.‐Y.C. wrote the paper and all authors reviewed the paper.

## Supporting information



Supporting Information

## Data Availability

The data that support the findings of this study are available from the corresponding author upon reasonable request.
