# Supplementary material for: RNA N 6‐Methyladenosine‐Binding Protein YTHDFs Redundantly Attenuate Cancer Immunity by Downregulating IFN‐γ Signaling in Gastric Cancer
Source: Adv Sci (Weinh). 2024 Nov 25;12(3):2410806. doi: 10.1002/advs.202410806 (PMC11744580; doi:10.1002/advs.202410806)
Supplement: Supplementary file 1 — Supporting Information [file ADVS-12-2410806-s001.pdf]

ADVANCED SCIENCE
Open Access

Supporting Information

for *Adv. Sci.*, DOI 10.1002/advs.202410806

RNA N^6-Methyladenosine-Binding Protein YTHDFs Redundantly Attenuate Cancer Immunity by Downregulating IFN-γ Signaling in Gastric Cancer

Dongjun Jang, Chanwoong Hwa, Seoyeon Kim, Jaeik Oh, Seungjae Shin, Soo-Jin Lee, Jiwon Kim, Sang Eun Lee, Yoojin Yang, Dohee Kim, Seoho Lee, Hae Rim Jung, Yumi Oh, Kyunggon Kim, Hye Seung Lee, Joon-Yong An and Sung-Yup Cho**

Supporting Information

RNA *N*^{6}-methyladenosine-binding protein YTHDFs redundantly attenuate cancer immunity by downregulating IFN-γ signaling in gastric cancer

Dongjun Jang, Chanwoong Hwa, Seoyeon Kim, Jaeik Oh, Seungjae Shin, Soo-Jin Lee, Jiwon Kim, Sang Eun Lee, Yoojin Yang, Dohee Kim, Seoho Lee, Hae Rim Jung, Yumi Oh, Kyunggon Kim, Hye Seung Lee, Joon-Yong An, Sung-Yup Cho**

Supplementary Figure S1

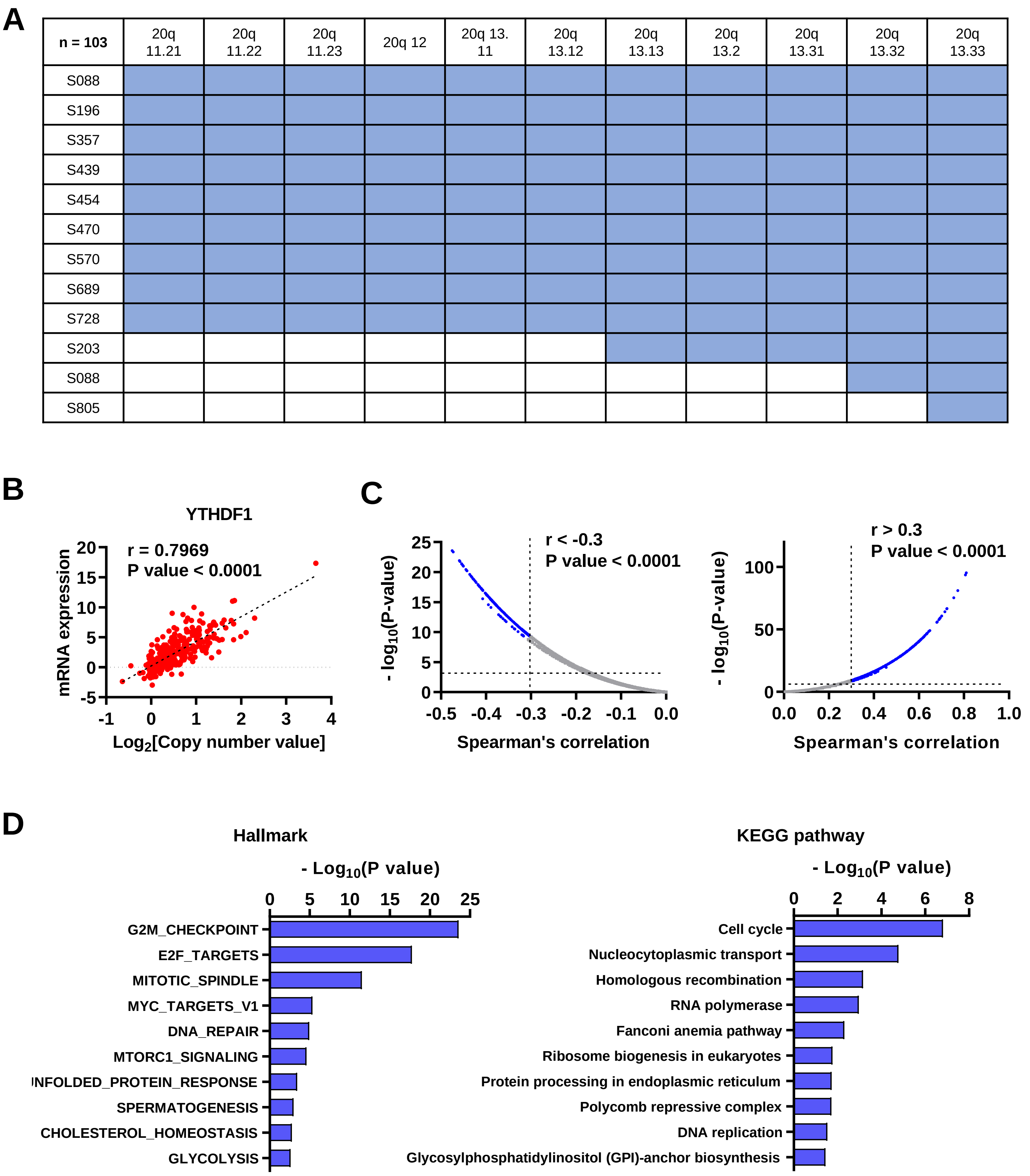

Supplementary Figure S1. Genomic alterations and mRNA expressions of YTHDF1 in gastric cancer (GC). A) The chromosomal locations of YTHDF1-containing amplicons in 12 YTHDF1-amplified patients estimated by array Comparative Genomic Hybridization (aCGH). B) Scatter plot displaying the correlation of DNA copy numbers from GISTIC 2.0 and mRNA expressions from RNA sequencing for YTHDF1 in TCGA GC cohort. Spearman's correlation coefficient (r) and P value of correlation are shown. C) Scatter plot displaying the correlation of Spearman's correlation coefficient (r) and P values of correlation for 642 genes that exhibited a negative correlation (left) and 1004 genes that exhibited a positive correlation (right) with YTHDF1 mRNA expression levels in TCGA GC cohort ($|\text{Spearman's correlation } r| > 0.3$, $P < 0.0001$). D) The top 10 hallmark pathway (left) and KEGG pathway gene sets (right) that were significantly enriched in the 1004 genes that exhibited a positive correlation with YTHDF1 mRNA expression levels are shown.

Supplementary Figure S2

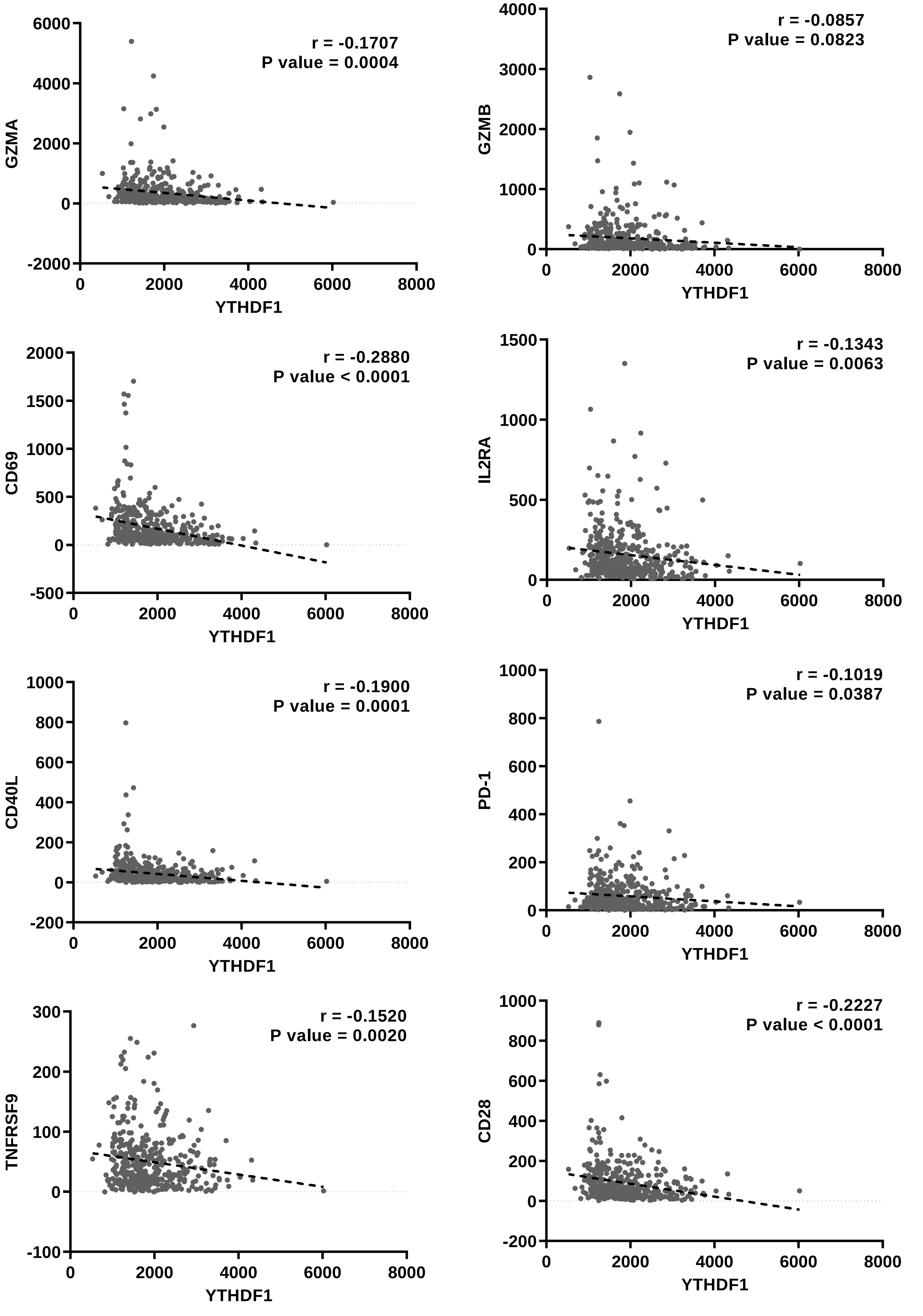

Supplementary Figure S2. Correlation analysis of YTHDF1 mRNA expression with immune-related genes. Scatter plot displaying the correlation of YTHDF1 mRNA expression with GZMA, GZMB, CD69, IL2A, CD40L, PD-L1, TNFRSF9 or CD28 mRNA expression in TCGA GC cohort. Spearman's correlation coefficients (r) and P values of correlation are shown.

Supplementary Figure S3

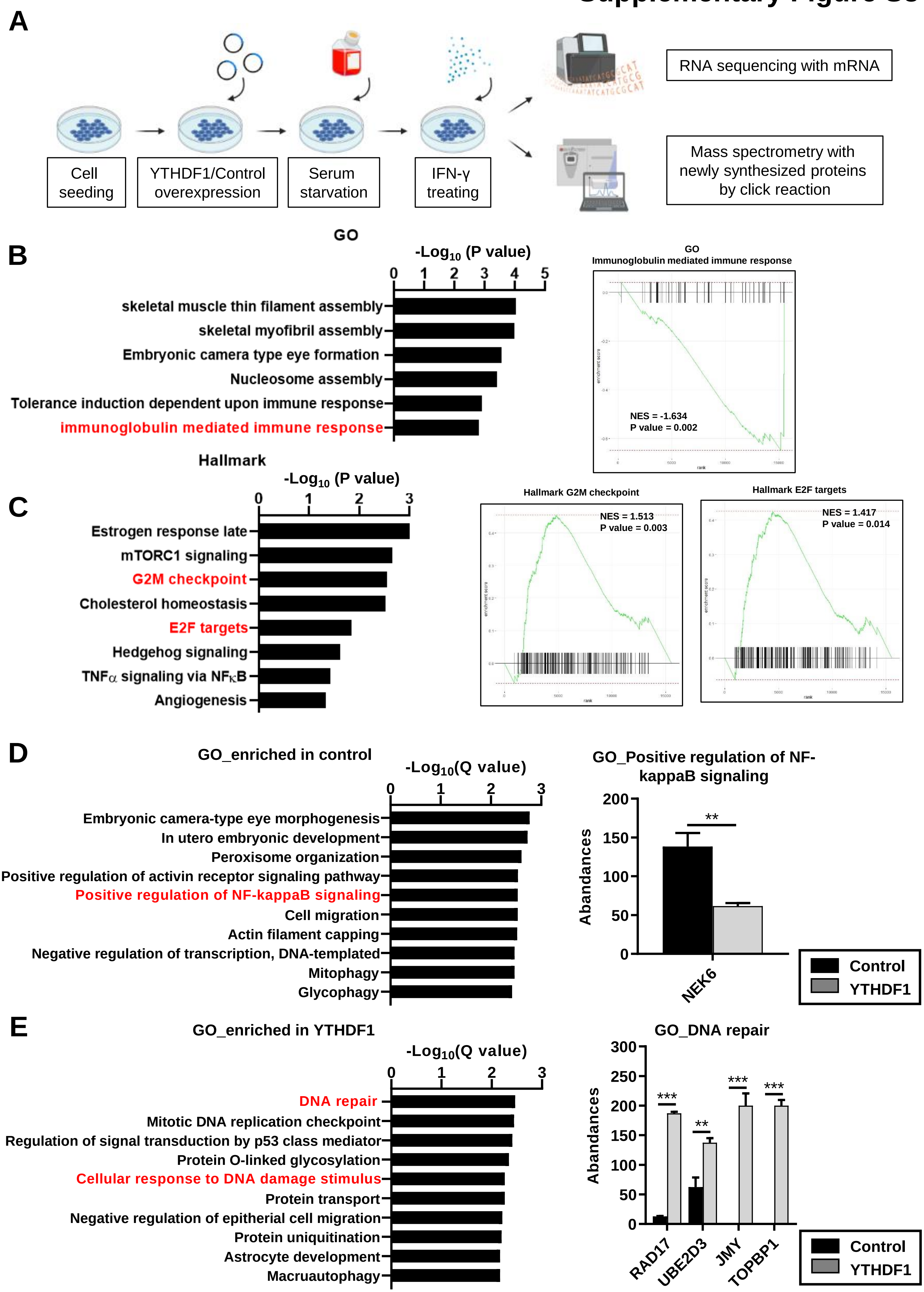

Supplementary Figure S3. Enriched gene sets by YTHDF1 overexpression in transcriptomic and proteomic analyses. A) Schematic of the RNA sequencing and proteomic analysis procedure: after overexpressing YTHDF1, SNU638 cells were serum-starved and treated with IFN-γ at 10 ng/ml for 24 h. The mRNA expressions were analyzed by RNA sequencing and newly synthesized proteins were evaluated by click reaction and mass spectrometry. B) Depleted gene ontology (GO) gene sets in YTHDF1-overexpressed SNU638 cells with IFNγ treatment by gene set enrichment analysis (GSEA) for RNA sequencing. The left graph shows the significantly depleted GO gene sets in YTHDF1-overexpressed cells ($P < 0.05$). The right graph shows the enrichment plot of a representative gene set ('Immunoglubulin mediated immune response') that was significantly depleted in YTHDF1-overexpressed cells. On the x-axis, genes are ranked from the most upregulated to the most downregulated between YTHDF1-overexpressed (left end; positively correlated) and control (right end; negatively correlated) cells. The y-axis shows a running enrichment score for YTHDF1-overexpression. C) Enriched hallmark gene sets in YTHDF1-overexpressed SNU638 cells with IFNγ treatment by GSEA for RNA sequencing. The left graph shows the significantly enriched gene sets in YTHDF1-overexpressed cells ($P < 0.05$). The right graphs show the enrichment plot of representative gene sets ('G2M checkpoint' and 'E2F Targets') that was significantly enriched in YTHDF1-overexpressed cells. On the x-axis, genes are ranked from the most upregulated to the most downregulated between YTHDF1-overexpressed (left end; positively correlated) and control (right end; negatively correlated) cells. The y-axis shows a running enrichment score for YTHDF1-overexpression. D) Enriched GO gene sets for differentially expressed proteins (DEPs) down-regulated by YTHDF1 overexpression. The left graph shows the significantly depleted GO gene sets in YTHDF1-overexpressed cells analyzed by DAVID (top 10 gene sets). The right graph shows protein abundance of NEK6 in SNU638 cells, which was measured by mass spectrometry using three independent biological replicates. (right). P value was calculated using unpaired t-test (**$P < 0.01$). E) Enriched GO gene sets for differentially expressed proteins (DEPs) up-regulated by YTHDF1 overexpression. The left graph shows the significantly enriched GO gene sets in YTHDF1-overexpressed cells analyzed by DAVID (top 10 gene sets). The right graph shows protein abundance of RAD17, UBE2D3, JMY and TOPBP1 in SNU638 cells, which was measured by mass spectrometry using three independent biological replicates. (right). P value was calculated using unpaired t-test (**$P < 0.01$, ***$P < 0.001$).

Supplementary Figure S4

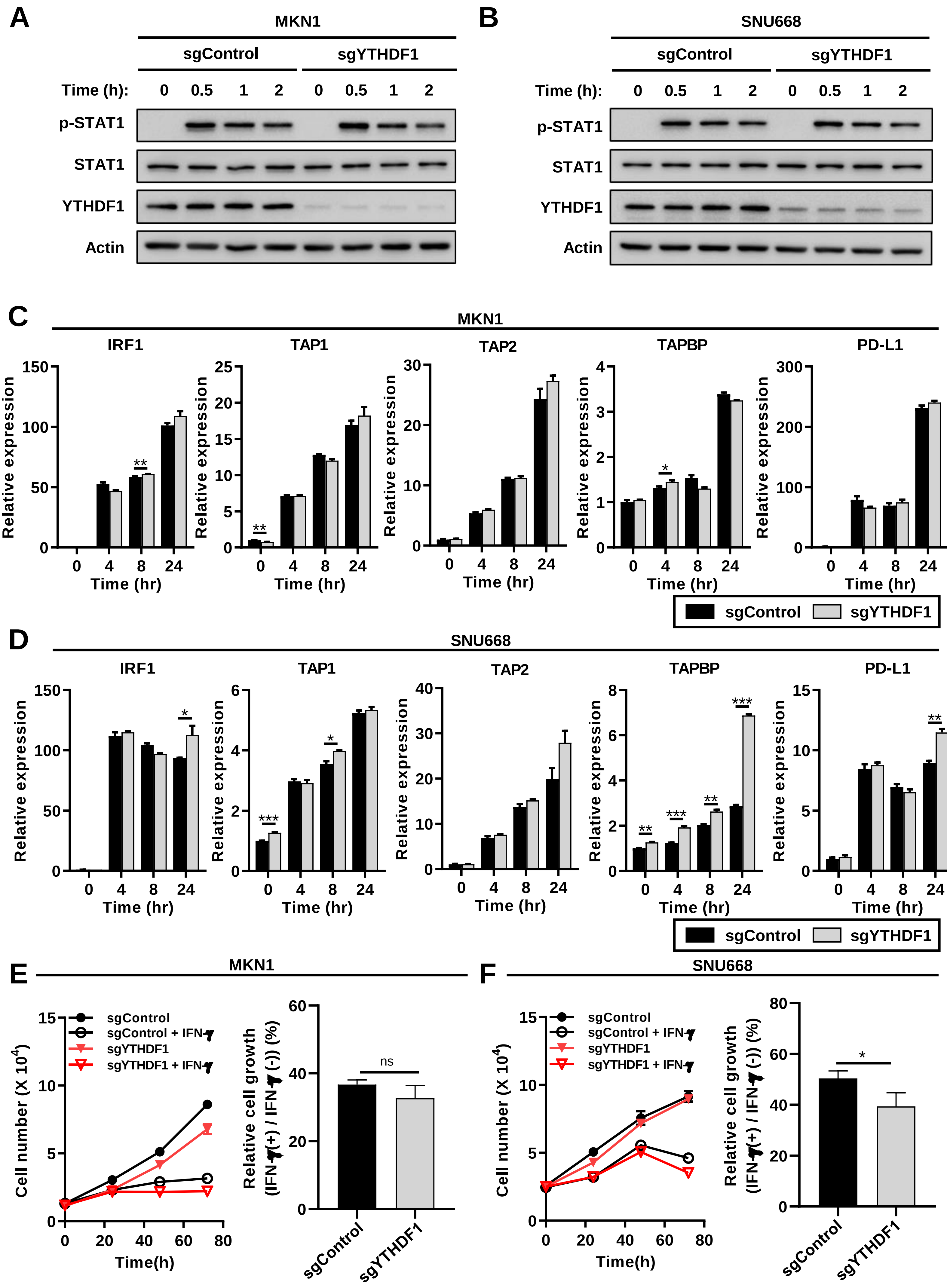

Supplementary Figure S4. Effect of YTHDF1 knock-down on IFN-γ response in MKN1 and SNU668 cells. A, B) Phosphorylation of STAT1 in YTHDF1 knock-down GC cells. After serum starvation for 24 h, MKN1 (A) and SNU668 (B) cells were treated with 1 ng/ml IFNγ for indicated time. The phosphorylation levels of STAT1 were evaluated by western blotting. sgControl: control single guide RNA, sgYTHDF1, single guide RNA for YTHDF1. C, D) Expression of IFNγ-responsive genes in YTHDF1 knock-down GC cells. YTHDF1 knock-down cell lines were generated using CRISPR/Cas9 with control sgControl and sgYTHDF1 in MKN1 (C) and SNU668 (D) cells. After serum starvation for 24 h, cells were treated with 10 ng/ml IFNγ and the mRNA expression levels of IFNγ-responsive genes were estimated by real-time PCR relative to the levels of GAPDH (n = 3) at indicated time points. Relative values are compared to 0 h in control. P value was calculated using unpaired t-test (*P < 0.05, **P < 0.01, ***P < 0.001). E, F) Suppression of cell proliferation by IFNγ treatment in YTHDF1 knock-down GC cells. After serum starvation for 24 h, MKN1 (E) and SNU668 (F) cells were treated with 10 ng/ml IFNγ for indicated time points and the cell numbers were estimated by trypan blue staining assay. Relative cell growth is estimated compared to each IFN-γ-untreated control in 72 h (right). P value was calculated using unpaired t-test (*P < 0.05).

Supplementary Figure S5

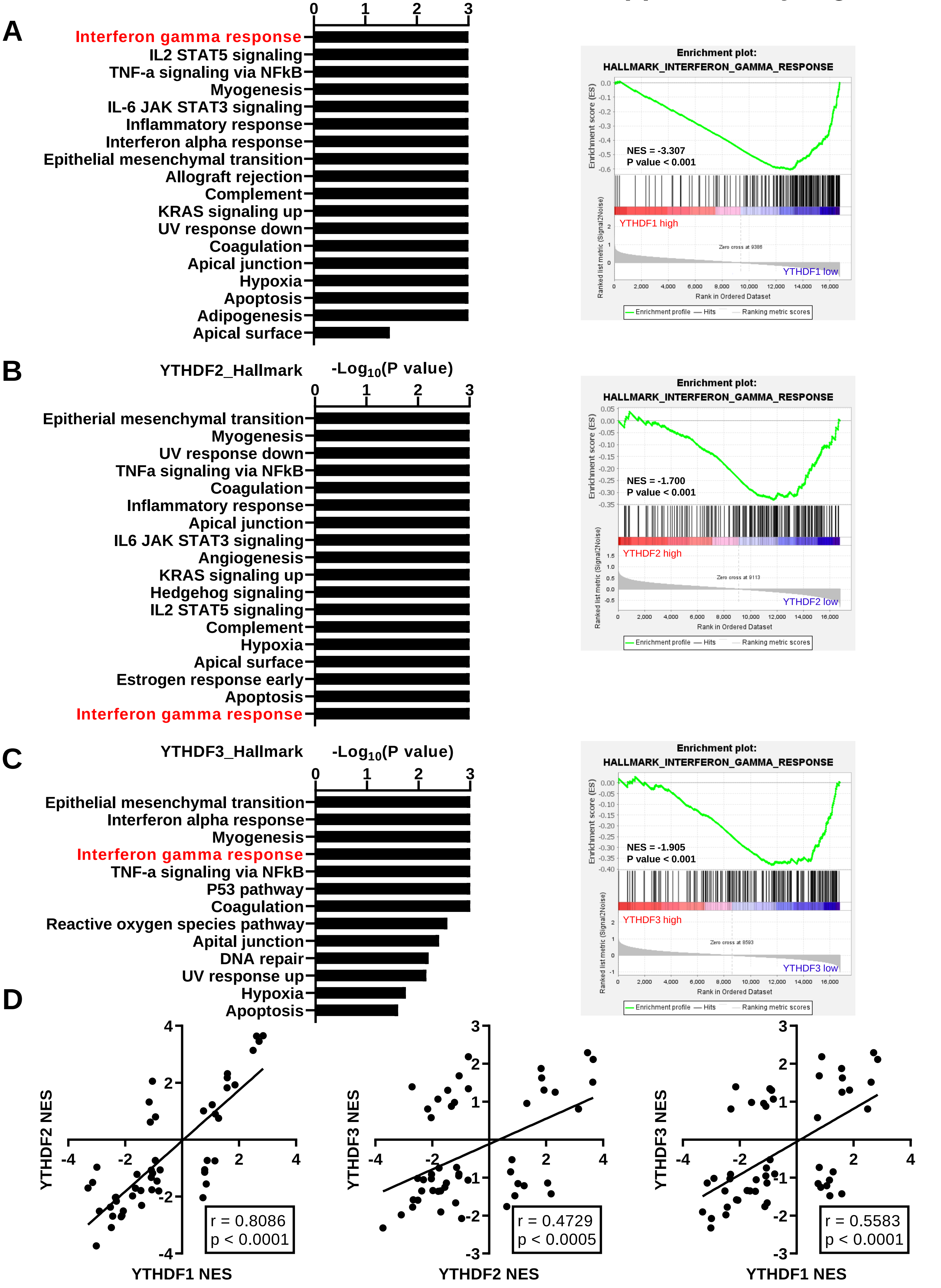

Supplementary Figure S5. Enriched hallmark gene sets in gastric cancer (GC) patient samples with low expression of YTHDF1-3. A, B, C) Enriched gene hallmark gene sets in GC patients with low expression of YTHDF1 (A), YTHDF2 (B), and YTHDF3 (C). The RNA sequencing data of GC patients were downloaded from TCGA pan-cancer database, and GSEA was performed between samples with the top 10 % and bottom 10% of YTHDF mRNA expression. The left graphs show the significantly enriched hallmark gene sets in patients with low expression of YTHDFs ($P < 0.05$, $Q < 0.25$). The right graphs show the enrichment plots of 'Interferon_gamma_response' gene set for each YTHDF1 expression. On the x-axis, genes are ranked from the most upregulated to the most downregulated between samples with YTHDFs high expression (left end; positively correlated) and low expression (right end; negatively correlated). The y-axis shows a running enrichment score for samples with YTHDF1 high expression. D) Scatter plots illustrating the correlation of normalized enrichment scores (NESs) for the YTHDF1-YTHDF2 pair (left), YTHDF2-YTHDF3 pair (middle), and YTHDF1-YTHDF3 pair (right). Spearman's correlation coefficient (r) and P value of correlation are shown.

Supplementary Figure S6

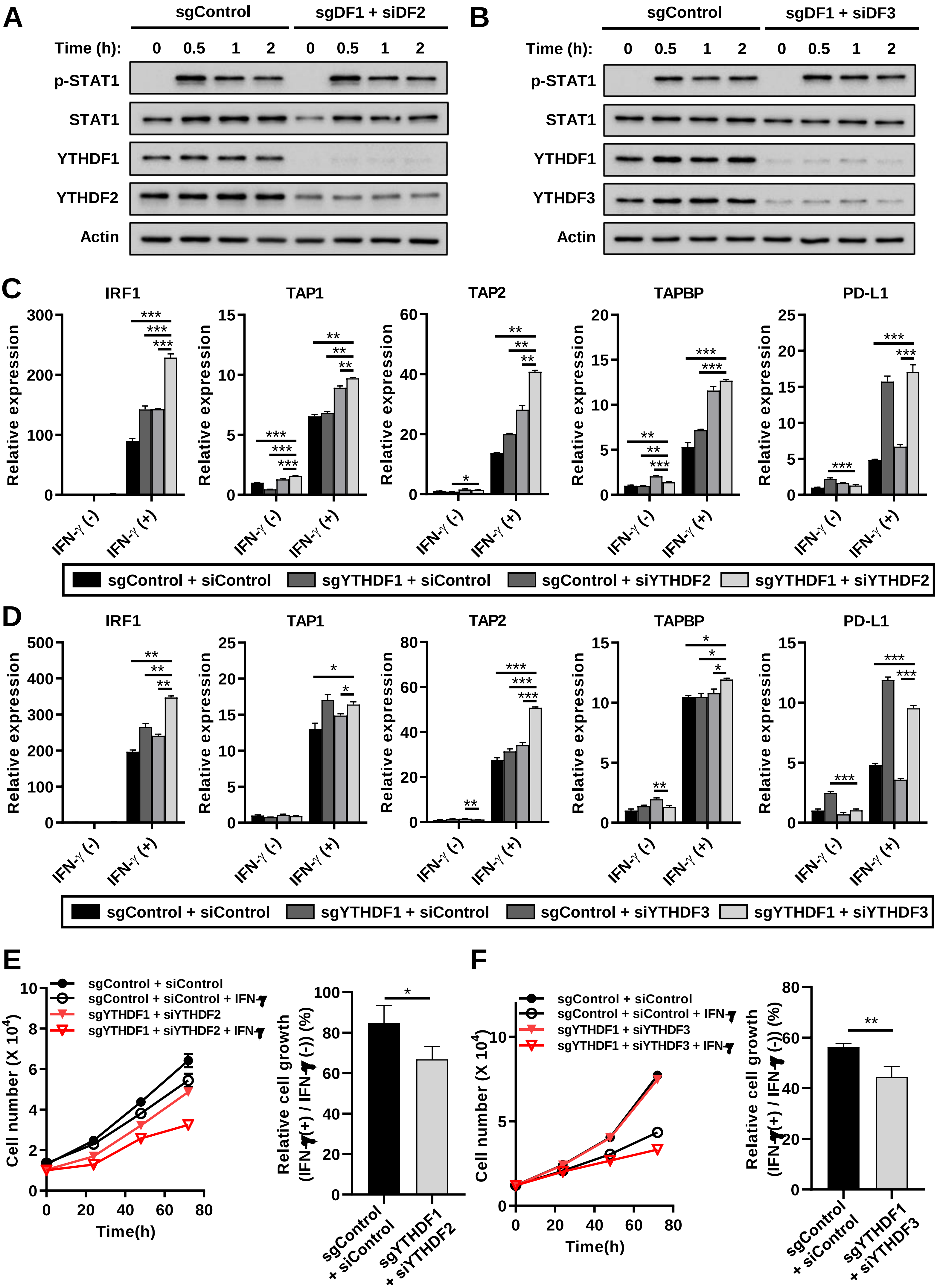

Supplementary Figure S6. The knock-down of YTHDFs synergistically enhances the IFN-γ response in SNU668 cells. A, B) Phosphorylation of STAT1 in YTHDF1-3 knock-down SNU668 cells. YTHDF1 was stably knocked down in SNU668 cells using the CRISPR/Cas9 method (sgDF1). Then, these cells were transfected with either siRNA targeting YTHDF2 (A) or siRNA targeting YTHDF3 (B) to further downregulate YTHDF2 or YTHDF3 expression. After serum starvation for 24 h, cells were treated with 1 ng/ml IFNγ for indicated time. The phosphorylation levels of STAT1 were evaluated by western blotting. sgControl: control single guide RNA, siControl : control siRNA, sgDF1: single guide RNA for YTHDF1, siDF2: siRNA for YTHDF2, siDF3: siRNA for YTHDF3. C, D) Expression of IFNγ-responsive genes in YTHDF1-3 knock-down SNU668 cells. YTHDF1 stably knocked down SNU668 cells were transfected with either siRNA targeting YTHDF2 (C) or siRNA targeting YTHDF3 (D) to further downregulate YTHDF2 or YTHDF3 expression. After serum starvation for 24 h, cells were treated with 10 ng/ml IFNγ and the mRNA expression levels of IFNγ-responsive genes were estimated by real-time PCR relative to the levels of GAPDH (n = 3) at indicated time points. Relative values are compared to 0 h in control. P values were calculated using one-way ANOVA (*P < 0.05, **P < 0.01, ***P < 0.001). E, F) Suppression of cell proliferation by IFNγ treatment in YTHDF1-3 knock-down GC cells. YTHDF1 stably knocked down SNU668 cells were transfected with either siRNA targeting YTHDF2 (E) or siRNA targeting YTHDF3 (F) to further downregulate YTHDF2 or YTHDF3 expression. After serum starvation for 24 h, cells were treated with 10 ng/ml IFNγ for indicated time points and the cell numbers were estimated by trypan blue staining assay. Relative cell growth is estimated compared to each IFN-γ-untreated control in 72 h (right). P values were calculated using unpaired t-test (*P < 0.05, **P < 0.01).

Supplementary Figure S7

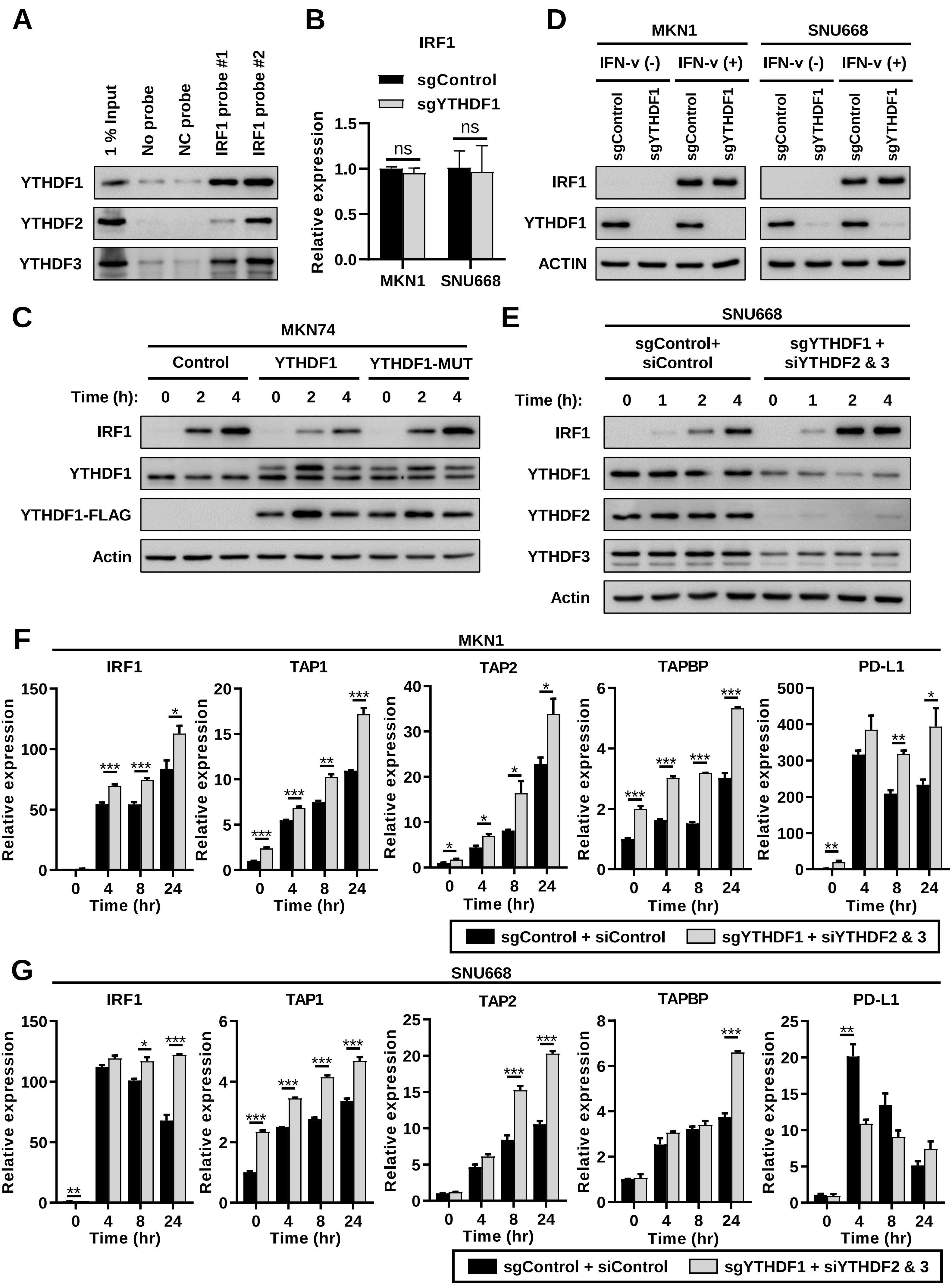

Supplementary Figure S7. Effect of YTHDFs regulation on IFN-γ response in MKN1 and SNU668 cells. A) The interaction between YTHDFs protein and IRF1 mRNA by RNA pull-down assay. Western blot images represent YTHDF1-3 protein levels in pull-down assays using each biotinylated probe after IFN-γ treatment (10 ng/ml) in MKN1 cells (No probe: without biotinylated probe; NC probe: biotinylated poly $(A)_{25}$ probe for negative control; IRF1 probe: biotinylated probe for IRF1). B) Effect of YTHDF1 knock-down on the newly synthesized mRNA levels of IRF1 in the presence of IFN-γ. YTHDF1 knock-down cell lines were generated using CRISPR/Cas9 with control single guide RNA (sgControl) and single guide RNA for YTHDF1 (sgYTHDF1) in MKN1 (left) and SNU668 (right) cells. After serum starvation for 24 h, cells were treated with 10 ng/ml IFNγ and the nascent RNA expression levels of IRF1 mRNA was estimated by 5-ethynyl uridine labeling and real-time PCR (n = 3). P values were calculated using unpaired t-test. C) Effect of YTHDF1 overexpression on the protein levels of IRF1 in the presence of IFN-γ. Western blot images represent IRF1 protein levels induced by IFN-γ treatment (1 ng/ml) in YTHDF1 or YTHDF1-MUT-overexpression MKN74. D) Effect of YTHDF1 knock-down on the protein levels of IRF1 in the presence of IFN-γ. Western blot images represent IRF1 protein levels induced by IFN-γ (1 ng/ml) in MKN1 (left) and SNU668 (right) cells with CRISPR-based YTHDF1 knock-down (sgControl: control sgRNA; sgYTHDF1: sgRNA for YTHDF1). E) Effect of YTHDF1-3 knock-down on the protein levels of IRF1 in the presence of IFN-γ. Western blot images represent IRF1 protein levels induced by IFN-γ (1 ng/ml) in SNU668 cells were transfected both siRNA targeting YTHDF2 and siRNA targeting YTHDF3 in CRISPR-based YTHDF1 knock-down (sgControl: control sgRNA; sgYTHDF1: sgRNA for YTHDF1; siControl: control siRNA, sgDF1: single guide RNA for YTHDF1, siDF2: siRNA for YTHDF2, siDF3: siRNA for YTHDF3.). F, G) Expression of IFNγ-responsive genes in YTHDF1-3 knock-down GC cells. YTHDF1 stably knocked down MKN1 (F) and SNU668 (G) cells were transfected with both siRNA targeting YTHDF2 and YTHDF3 to further downregulate YTHDF2 and YTHDF3 expression. After serum starvation for 24 h, cells were treated with 10 ng/ml IFNγ and the mRNA expression levels of IFNγ-responsive genes were estimated by real-time PCR relative to the levels of GAPDH (n = 3) at indicated time points. Relative values are compared to 0 h in control. P values were calculated using unpaired t-test (*P < 0.05, **P < 0.01, ***P < 0.001).

Supplementary Figure S8

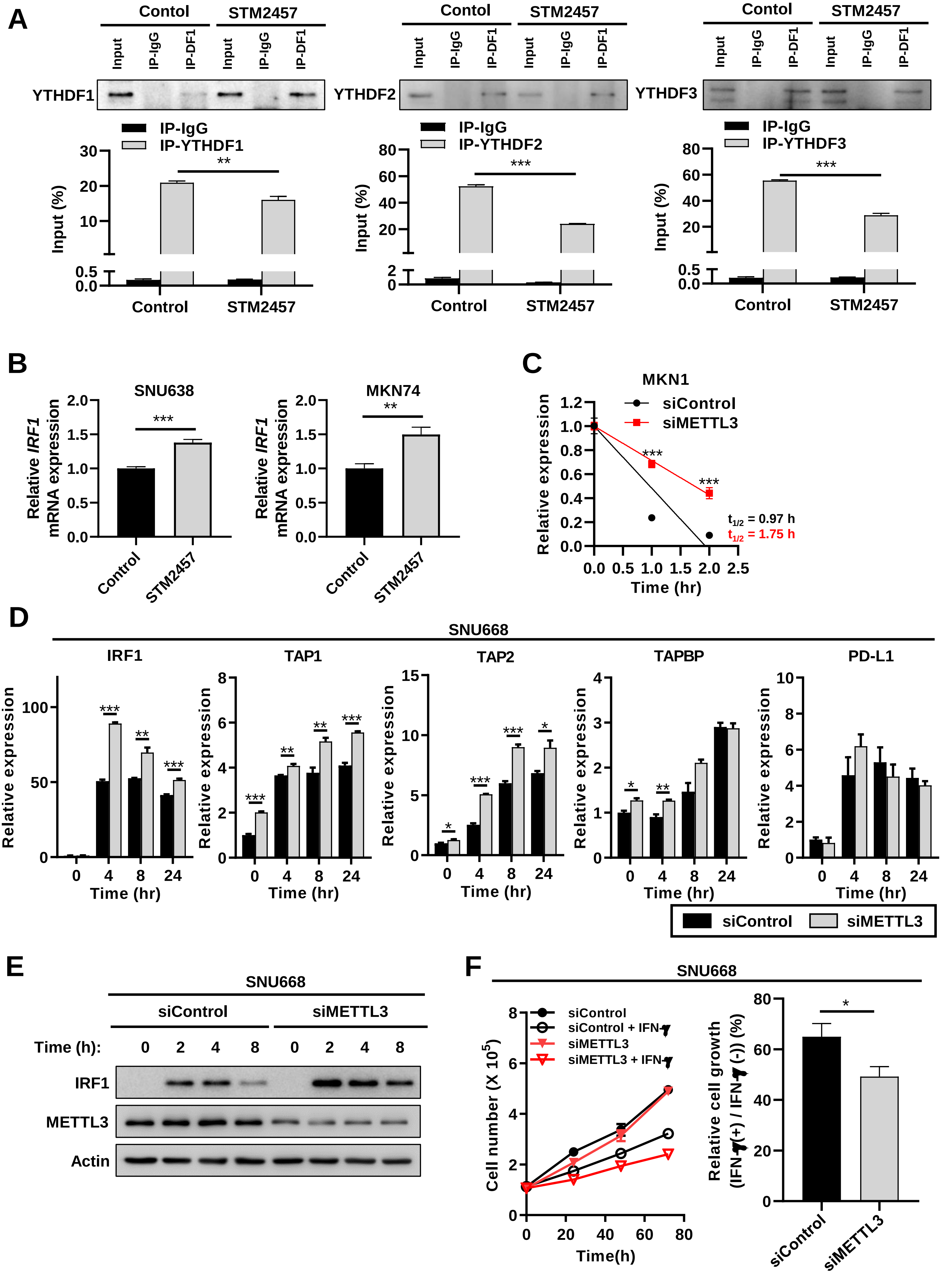

Supplementary Figure S8. The inhibition of METTL3 reduces the m6A modification of IRF1 mRNA in gastric cancer (GC) cells. A) Reduced binding of YHDF1-3 to IRF1 mRNA by STM2457 was validated through RIP-qPCR analysis. After MKN74 cells were pre-treated with 10 μM STM2457 for 1 h, cells were treated with 10 ng/ml IFNγ with 10 μM STM2457 for 24 h. Immunoprecipitation was performed using anti-YTHDF1, anti-YTHDF2, or anti-YTHDF3 antibody. P values were calculated using unpaired t-test (**P < 0.01, ***P < 0.001). B) Relative mRNA levels of IRF1 after treatment of STM2457 in GC. After SNU638 (left) and MKN74 (right) cells were pre-treated with 10 μM STM2457 for 1 h, cells were treated with 10 ng/ml IFNγ with 10 μM STM2457 for 24 h. The mRNA levels were determined by real-time PCR relative to the levels of GAPDH. Relative values are estimated compared to control. P values were calculated using unpaired t-test (**P < 0.01, ***P < 0.001). C) Effect of METTL3 knock-down in IRF1 mRNA stability. Relative IRF1 mRNA levels were determined by real-time PCR after YTHDF1 knock-down using siRNA targeting METTL3 in MKN1 cells. Serum-starved cells were treated with 10 ng/ml IFN-γ for 24 h, before treatment of actinomycin D (10 μg/ml). P values were calculated using unpaired t-test (***P < 0.001). D) Expression of IFNγ-responsive genes in METTL3 knock-down GC cells. SNU668 cells were transfected with siRNA targeting METTL3. After serum starvation for 24 h, cells were treated with 10 ng/ml IFNγ and the mRNA expression levels of IFNγ-responsive genes were estimated by real-time PCR relative to the levels of GAPDH (n = 3) at indicated time points. Relative values are compared to 0 h in control. P values were calculated using unpaired t-test (*P < 0.05, **P < 0.01, ***P < 0.001). E) Effect of METTL3 knock-down on the protein levels of IRF1 in the presence of IFN-γ. Western blot images represent IRF1 protein levels induced by IFN-γ (1 ng/ml) in SNU668 cells were transfected siRNA targeting METTL3 (siControl: control siRNA, siMETTL3: siRNA for METTL3). F) Suppression of cell proliferation by IFNγ treatment in METTL3 knock-down GC cells. After serum starvation for 24 h, SNU668 cells were treated with 10 ng/ml IFNγ for indicated time points and the cell numbers were estimated by trypan blue staining assay. Relative cell growth is estimated compared to each IFN-γ-untreated control in 72 h (right). P values were calculated using unpaired t-test (*P < 0.05).

Supplementary Figure S9

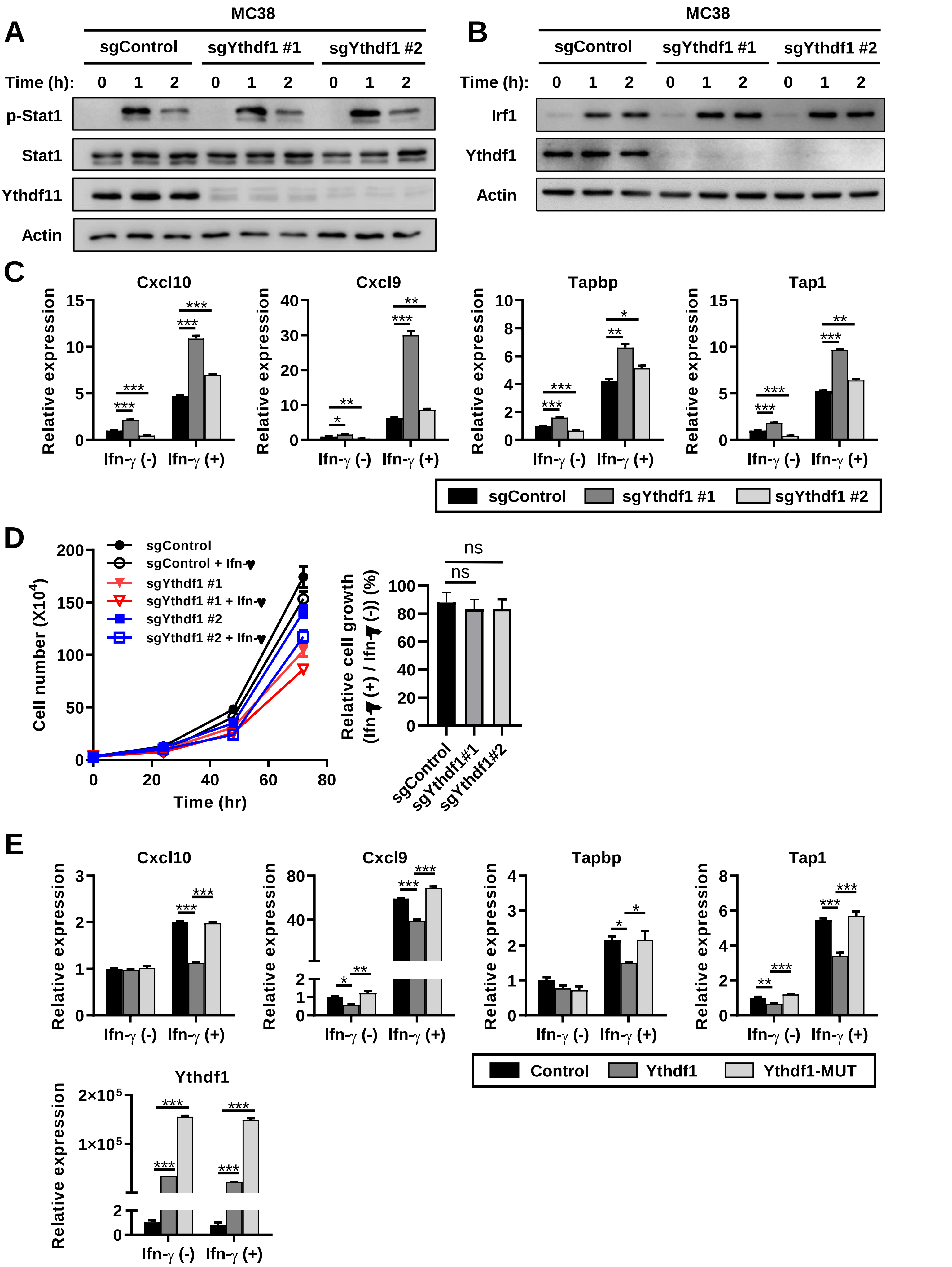

Supplementary Figure S9. Effect of Ythdf1 regulation on Ifn-γ response in mouse MC38 cells. A) Phosphorylation of Stat1 in Ythdf1 knock-down MC38 cells. After serum starvation for 24 h, MC38 cells were treated with 1 ng/ml mouse Ifnγ for indicated time. The phosphorylation levels of Stat1 were evaluated by western blotting. sgControl: control single guide RNA, sgYthdf1, single guide RNA for Ythdf1. B) Effect of Ythdf1 knock-down on the protein levels of Irf1 in the presence of Ifn-γ. Western blot images represent Irf1 protein levels induced by Ifn-γ (1 ng/ml) in MC38 with CRISPR-based Ythdf1 knock-down (sgControl: control sgRNA; sgYthdf1: sgRNA for Ythdf1). C) Expression of mouse Ifnγ-responsive genes in Ythdf1 knock-down MC38 cells. Relative mRNA levels of Cxcl10, Cxcl9, Tapbp and Tap1 induced by 10 ng/ml Ifn-γ after knock-down Ythdf1 in MC38. The mRNA levels were determined by real-time PCR relative to the levels of Gapth. Relative values are estimated compared to 0 h in control. P values were calculated using one-way ANOVA (*P < 0.05, **P <0.01, ***P < 0.001). D) Effect of Ythdf1 knock-down on *in vitro* proliferation of Ifn- γ-treated MC38 cells. Live cells were measured using the trypan blue staining assay after treatment with 10 ng/ml Ifn-γ for 0, 24, 48, and 72 h (left). Relative cell growth is presented compared to each IFN-γ-non-treated control in 72 h (right). E) Expression of Ifnγ-responsive genes in wild-type Ythdf1 or m6A-binding defective mutant Ythdf1 (Ythdf1-MUT)-overexpressed MC38 cells. Wild-type Ythdf1 or Ythdf1-MUT was overexpressed for 24 h in MC38 cells. After serum starvation for 24 h, cells were treated with 10 ng/ml Ifnγ and the mRNA expression levels of Ythdf1 and Ifnγ-responsive genes were estimated by real-time PCR relative to the levels of Gapdh (n = 3) at indicated time points. Relative values are compared to 0 h in control. P values were calculated using one-way ANOVA (*P < 0.05, **P < 0.01, ***P < 0.001).

Supplementary Figure S10

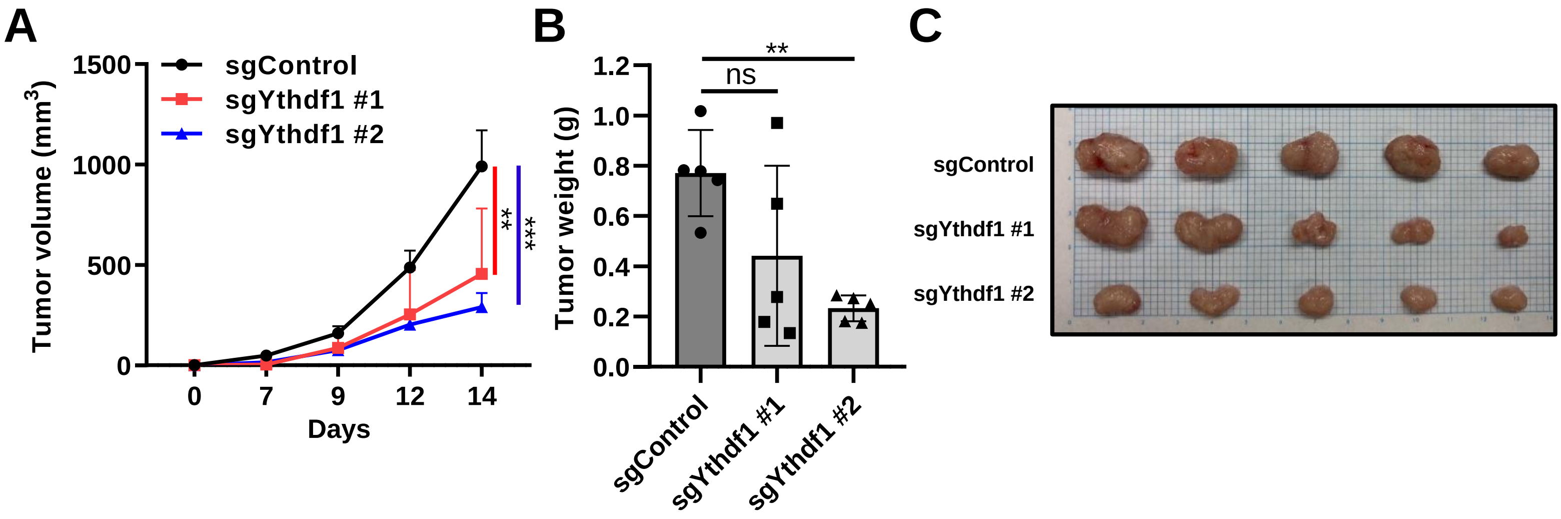

Supplementary Figure S10. Effect of Ythdf1 knock-down in mouse MC38 syngeneic mouse models. A) Tumor growth curves for subcutaneous control and YTHDF1 knockdown MC38 cells in NSG mice. Tumor volume measurements at indicated time points were calculated using the formula: 0.5 × (length × width^2). n = 5 mice per group (sgControl: control sgRNA; sgYthdf1: sgRNA for Ythdf1). P values were calculated using one-way ANOVA (**P < 0.01, ***P < 0.001). B, C) Tumor weight (B) and image (C) of control and YTHDF1 knock-down cell xenograft models (n = 5). P values were calculated using one-way ANOVA (**P < 0.01).

Supplementary Figure S11

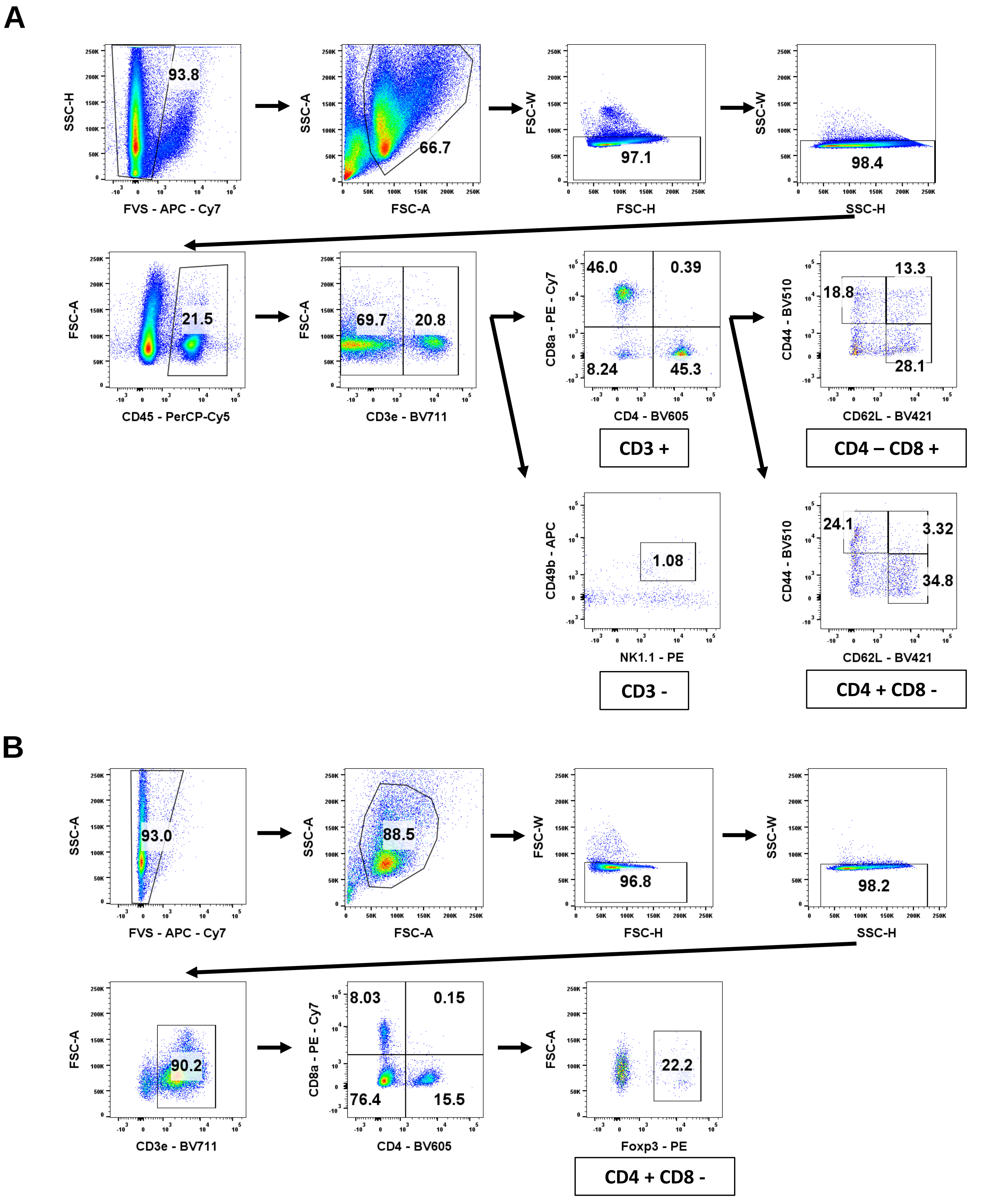

Supplementary Figure S11. Gating strategy operated in flow cytometry analysis to identify various subsets of lymphocytes. A, B) Gating strategy used in flow cytometry analysis to detect different subsets of mouse immune cells. Cells were gated on leukocyte population (CD 45^{+}), and each subset of immune cells was estimated according to immune cell marker signature.

Supplementary Figure S12

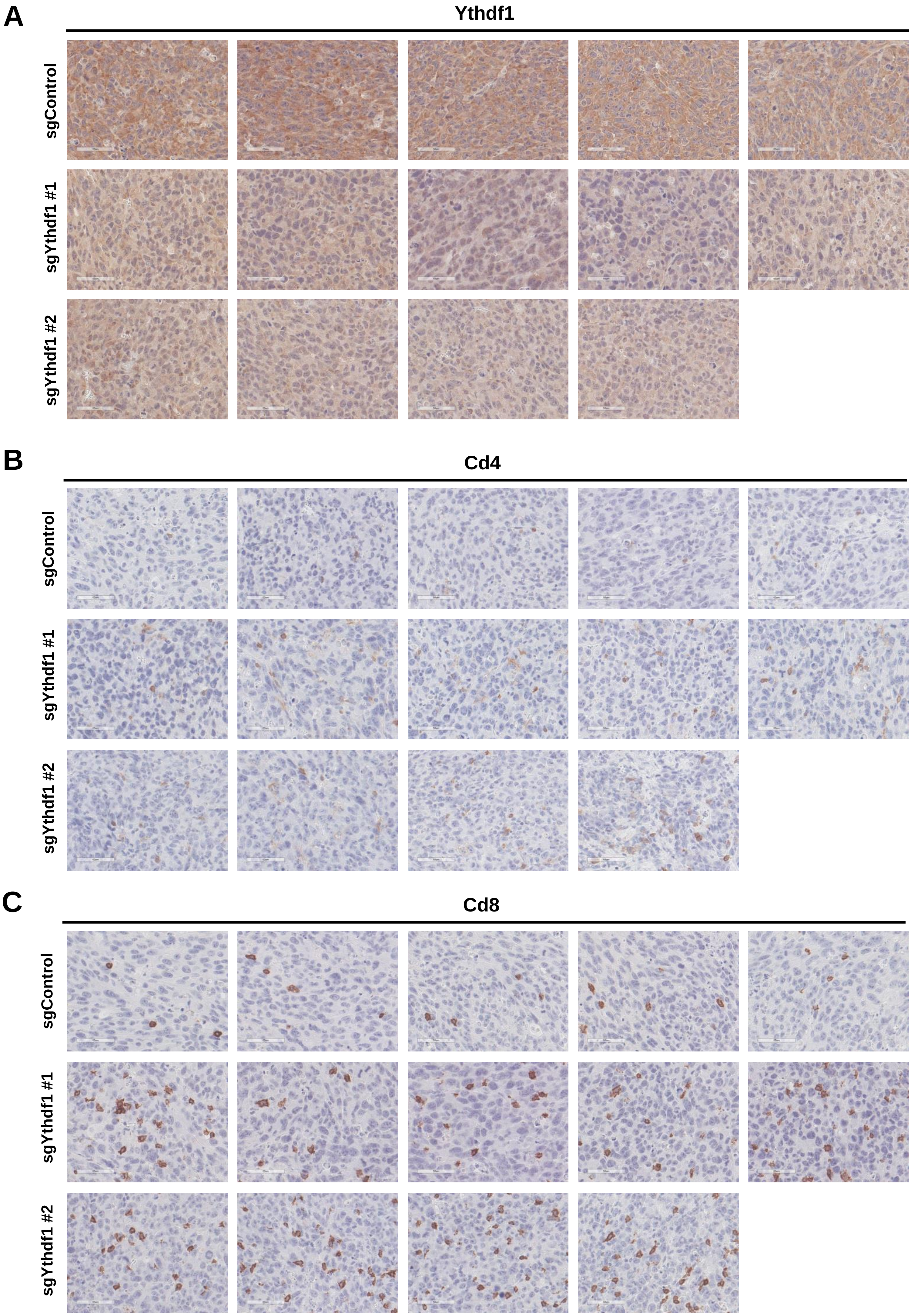

D

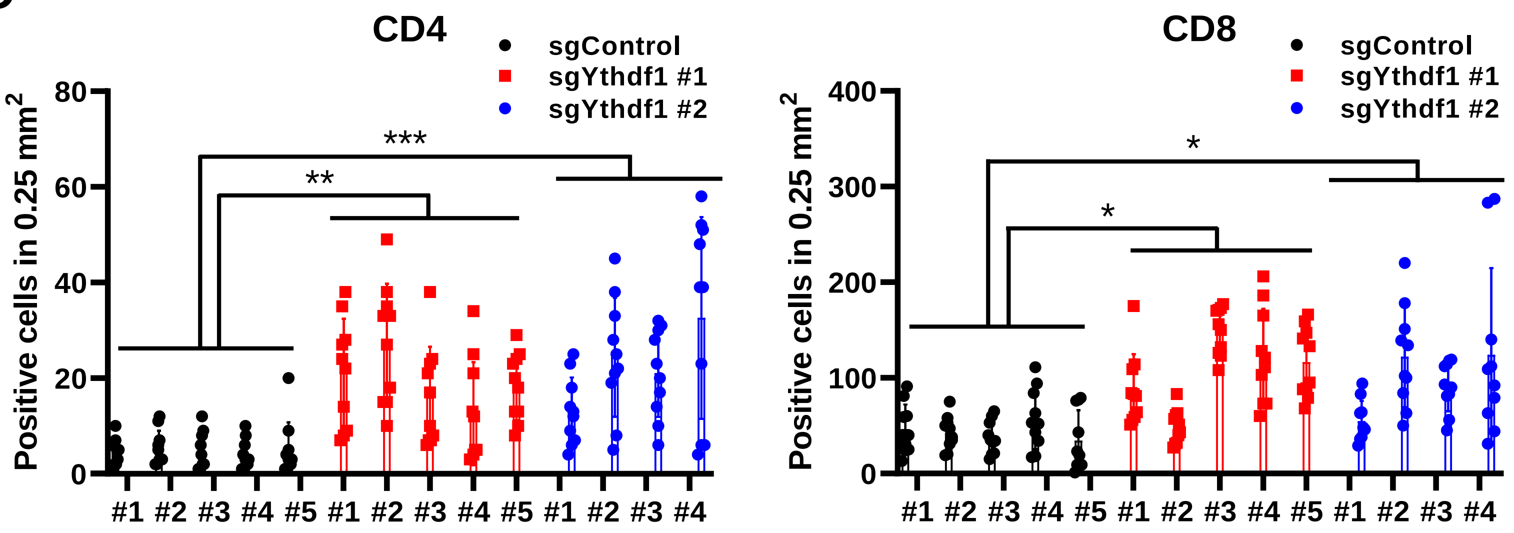

E

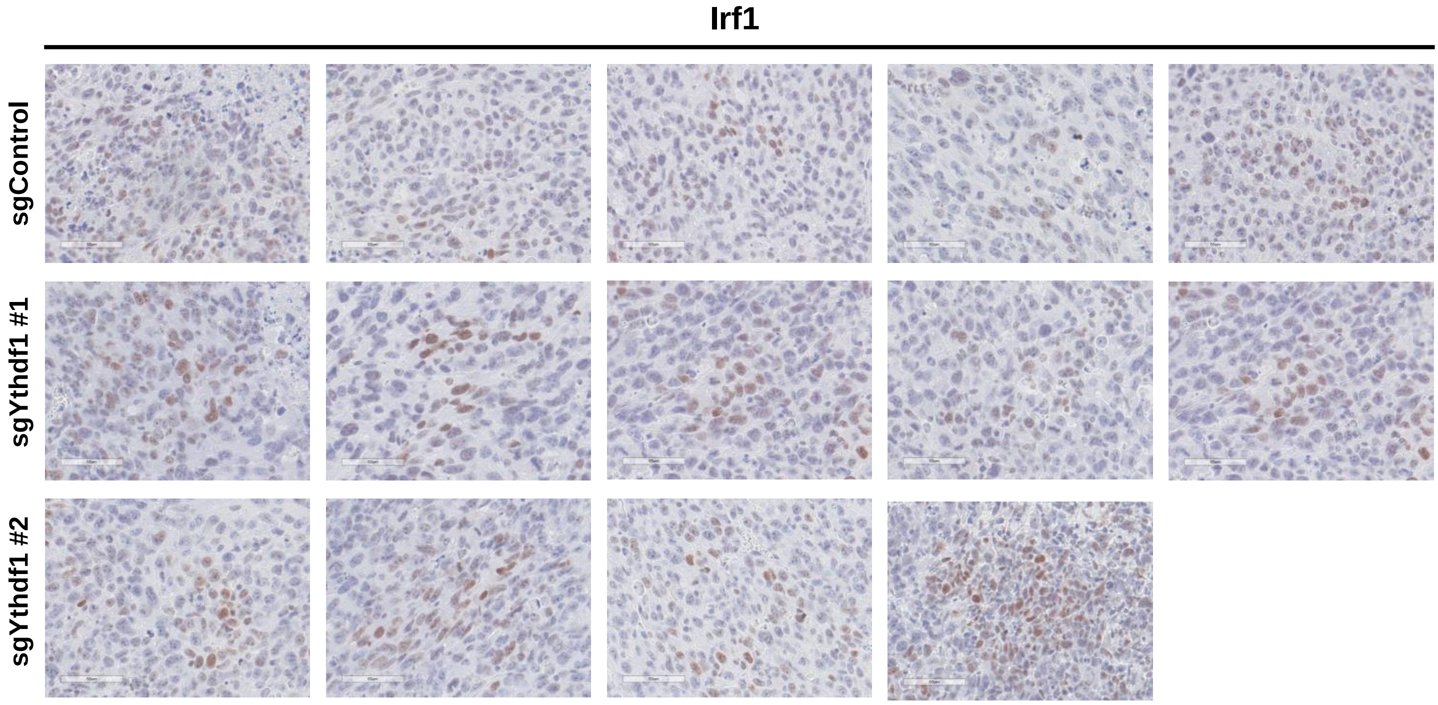

Supplementary Figure S12. Immunohistochemical staining of the Ythdf1, Cd4, Cd8 and Irf1 in Ythdf1 knock-down MC38 tumor. A, B, C, E) Immunohistochemical staining of α-Ythdf1 (A), α-Cd4 (B), α-Cd8 (C) and α-Irf1 (E) were determined. Representative photographs were showed. Scale bars: 50 μm. D) Immunohistochemical staining of α-Cd4 (left) and α-Cd8 (right) were determined the number of positive cells observed at 0.25 mm^2 was measured. P values were calculated using one-way ANOVA (*P < 0.05, **P < 0.01, ***P < 0.001). Scale bars: 50 μm. (n = 5 in every group).

Supplementary Figure S13

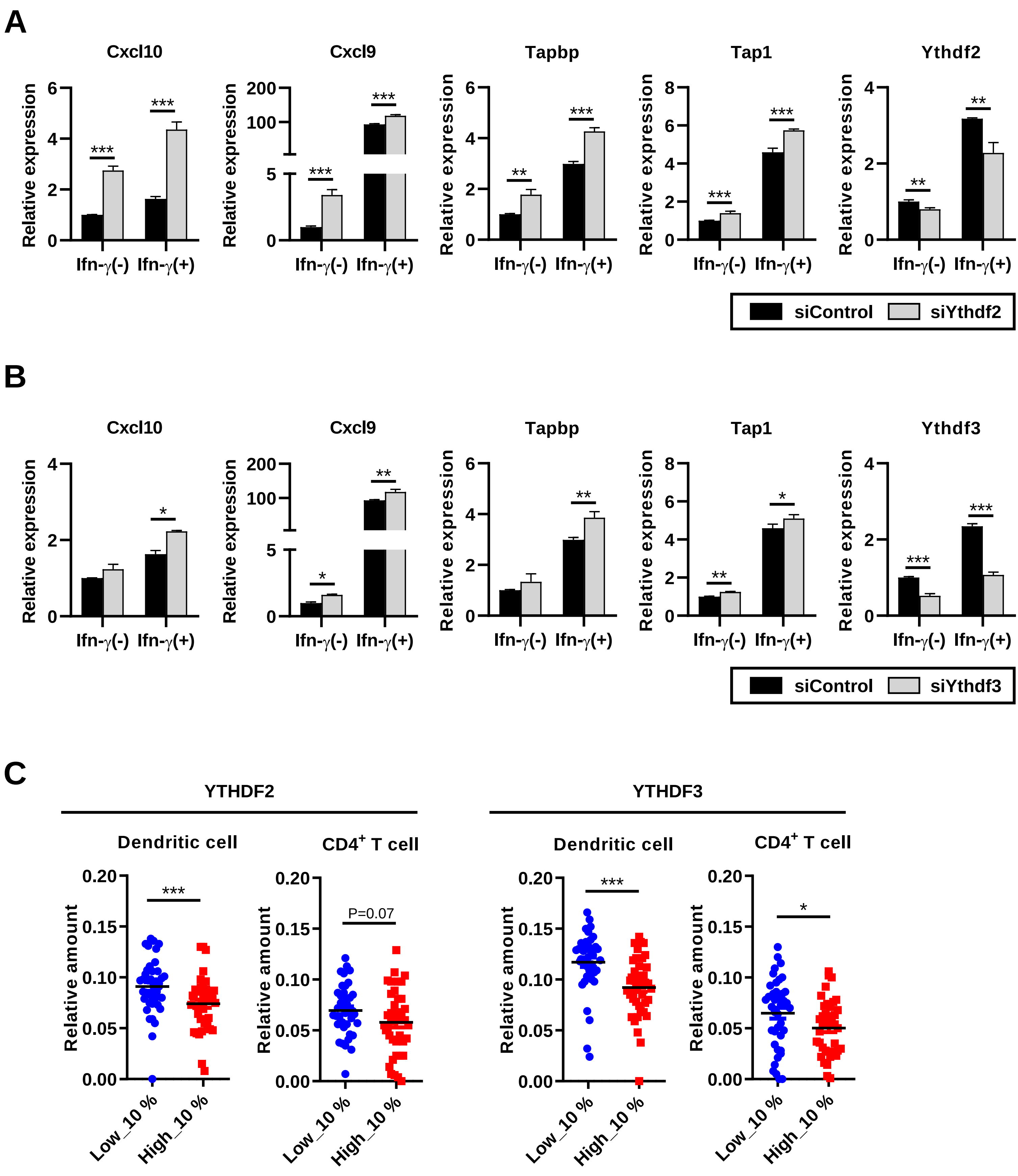

Supplementary Figure S13. Effect of Ythdf2 or Ythdf3 regulation on Ifn-γ response in mouse MC38 cells. A, B) Expression of Ifnγ-responsive genes in Ythdf2 (A) or Ythdf3 (B) knock-down MC38 cells. MC38 cells were transfected with siRNA targeting Ythdf2 or Ythdf3. After serum starvation for 24 h, cells were treated with 10 ng/ml Ifnγ and the mRNA expression levels of Ifnγ-responsive genes were estimated by real-time PCR relative to the levels of Gapdh (n = 3) at indicated time points. Relative values are compared to 0 h in control. P values were calculated using unpaired t-test (*P < 0.05, **P < 0.01, ***P < 0.001). C) The distribution patterns of dendritic cells and $CD4^+$ T cells according to YTHDF2 or YTHDF3 mRNA expressions. Relative amounts of each immune cell type analyzed by immuCellAI tool using 10 % patients (n = 41) with the highest and lowest expression of YTHDF2 or YTHDF3 mRNAs in TCGA GC cohort were demonstrated. P values were calculated using unpaired t-test (*P < 0.05, ***P < 0.001).

Supplementary Figure S14

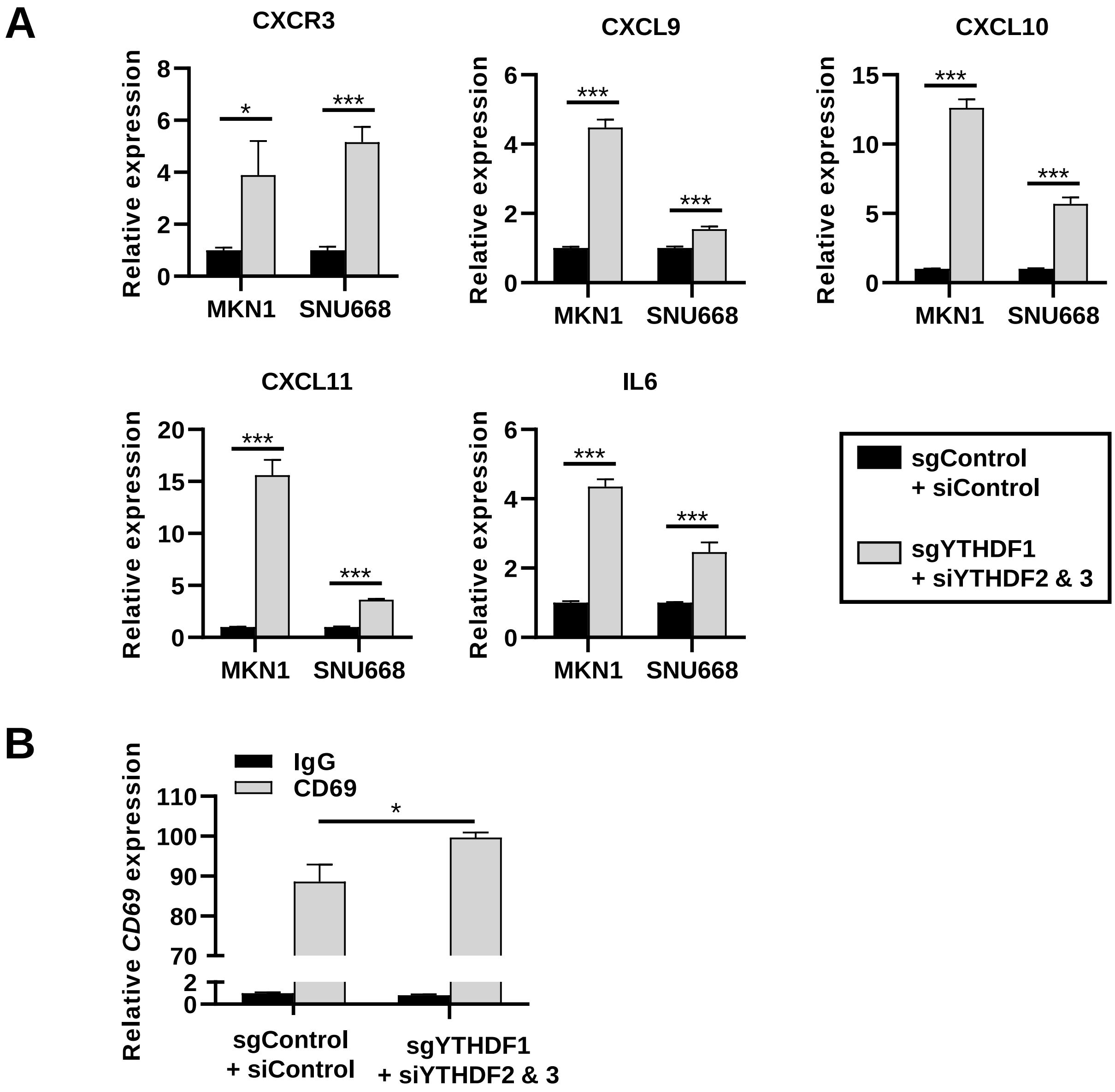

Supplementary Figure S14. The knock-down of YTHDFs in GC cells enhance the immune cell activation in vitro. A) Expression of IFNγ-responsive cytokine or receptor genes in YTHDF1-3 knock-down GC cells. Stable YTHDF1 knockdown MKN1 and SNU668 cells were transfected with siRNAs targeting YTHDF2 and YTHDF3 to further reduce YTHDF2 and YTHDF3 expression. After serum starvation for 24 h, cells were treated with 10 ng/ml IFNγ and the mRNA expression levels of IFNγ-responsive genes were estimated by real-time PCR relative to the levels of GAPDH (n = 3) at indicated time points. Relative values are compared to control cells. P values were calculated using unpaired t-test (*P < 0.05, ***P < 0.001). B) Reduced YTHDF1-3 expression in gastric cancer cells enhances the expression of the T-cell activation marker CD69 in co-cultured Jurkat cells. Jurkat cells were co-cultured with either control cells or CRISPR-based YTHDF1 knock-down SNU668 cells transfected with siRNAs targeting YTHDF2 and YTHDF3 in the presence of 10 ng/ml IFN-γ for 24 h. CD69 expression were measured by flow cytometry after anti-CD3/CD28 mouse IgG stimulation for 8 h. P values were calculated using unpaired t-test (*P < 0.05).

Supplementary Figure S15

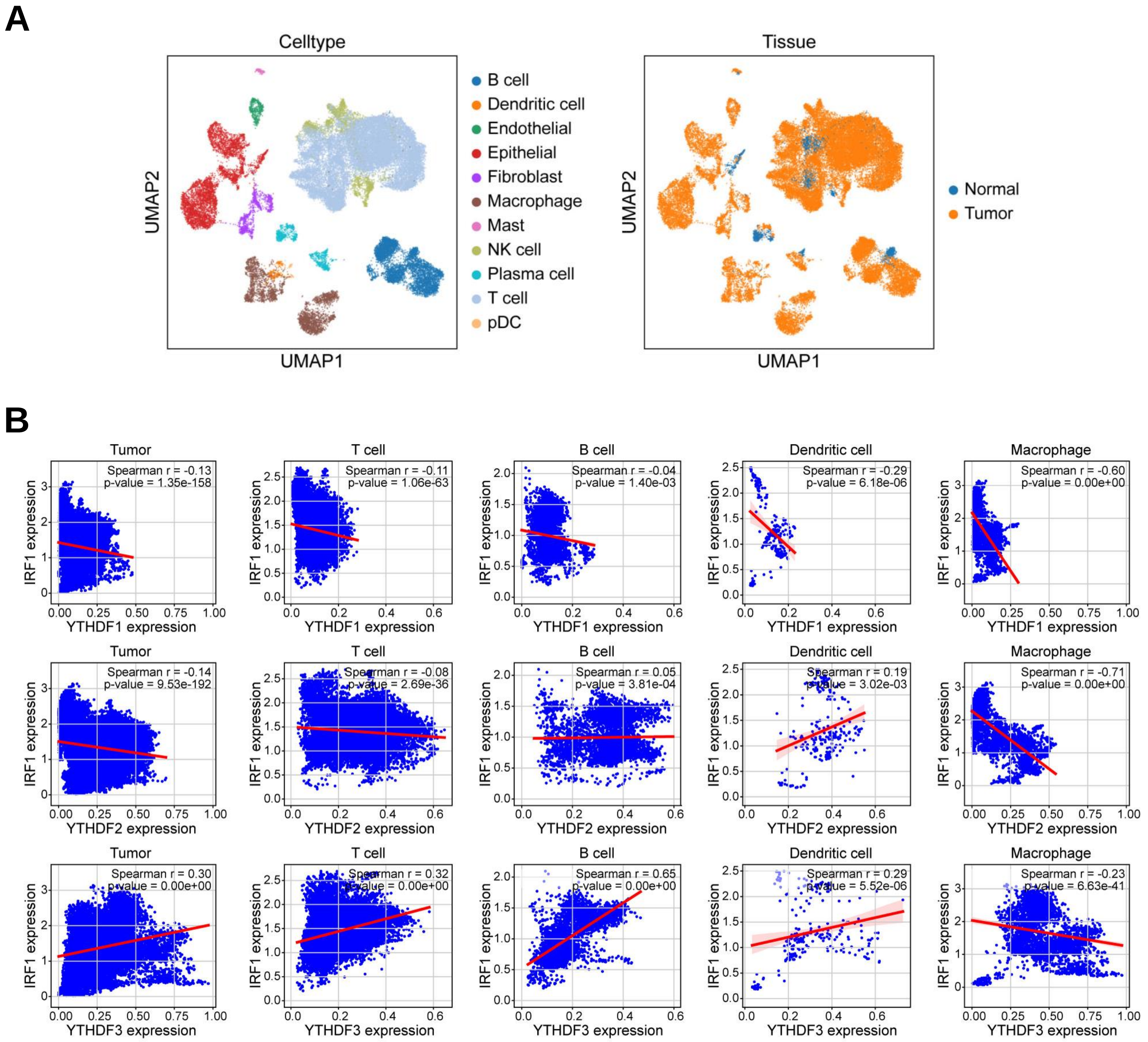

Supplementary Figure S15. Single cell RNA sequencing analysis from gastric cancer tissues. A) UMAP visualization of cell types in tumor tissue. UMAP projections illustrate the cellular and tissue distribution within the dataset. B) Correlation analysis of YTHDF proteins with IRF1 expression in tumor tissues. Scatter plots showing the relationship between the expression of YTHDF1, YTHDF2, and YTHDF3 with IRF1 across different cell types in tumor tissue. Each point represents a cell from imputed single-cell RNA-sequencing data, with blue dots indicating individual cell expression levels. Spearman correlation coefficients r and associated P values are displayed on each plot. Red lines represent the regression fits, highlighting the correlation trend.

Table S1. Primer list for real-time PCR.

Gene	5-Forward-3	5-Reverse-3
hIRF1	CTCTGAAGCTACAACAGATGAG	GTAGACTCAGCCCAATATCCC
hTAP1	AGGTACTGCTCTCCATCTAC	AGTGTAAGGGAGTCAACAGA
hTAP2	ACGGCTGAGCTCGGATACCAC	CCTCGGCCCCAAAACTGC
hTAPBP	ACCCTGGAGGTAGCAGGTCTTT	AATCCTTGCAGGTGGACAGGTAG
hPD-L1	GTGGCATCCAAGATACAAACTCAA	TCCTTCCTCTTGTCACGCTCA
hYTHDF1	CAAGCACACAACCTCCATCTTCG	GTAAGAAACTGGTTCGCCCTCAT
hCXCR3	TGCCAATACAACTTCCCACA	CGGAACTTGACCCCTACAAA
hCXCL9	CTGTTCCTGCATCAGCACCAAC	TGAACTCCATTCTTCAGTGTAGCA
hCXCL10	GTGGCATTCAAGGAGTACCTC	GCCTTCGATTCTGGATTCAGACA
hCXCL11	AAGGACAACGATGCCTAAATCCC	CAGATGCCCTTTTCCAGGACTTC
hIL-6	AGACAGCCACTCACCTCTTCAG	TTCTGCCAGTGCCTCTTTGCTG
hGAPDH	GAAGGTGAAGGTCGGAGT	GAAGATGGTGATGGGATTTC
mTap1	CTGGCAACCAGCTACGGGT	TGAGAAAGAGGATGTGGTGGG
mTapbp	ACAAGGCCCCCAGAGTGT	GGAAGAAGTGGGATGCAAGA
mCxcl9	GGAACCCTAGTGATAAGGAATGCA	TGAGGTCTTTGAGGGATTTGTAGTG
mCxcl10	TCCTTGTCCTCCCTAGCTCA	ATAACCCCTTGGGAAGATGG
mYthdf2	GGTTCTGTGCATCAAAAGGATGG	CCAAAGAATAGGAAAAGCCAATGG
mYthdf3	GGTTCGATTCATCAAAAAGATGCTG	GATCTGACATTGGTGGATAGCTG
mGapdh	TCACCACCATGGAGAAGGC	GCTAAGCAGTTGGTGGTGCA

Table S2. Correlation of YTHDF1 protein expression and clinical characteristics (YTHDF1 expressions in central tumor regions).

Characteristics	N = 400	YTHDF1 expression		P value
		High (N = 254)	Low (N = 146)	
**Gender**				< 0.0001
Male	255	180	75	
Female	145	74	71	
**Age (years)**				0.0271
< 60	212	124	88	
≥60	188	130	58	
**Lymphatic meta**				0.0067
No	122	65	57	
Yes	254	189	89	
**Vascular meta**				0.107
No	335	207	128	
Yes	65	47	18	
**Perineural meta**				0.2407
No	138	93	45	
Yes	262	161	101	
**MSI**				0.9989
0	337	214	123	
1~2	63	40	23	
**TNM 8th**				0.5677
1~2	187	116	71	
3	213	138	75	
**Preexisting_adenoma**				0.2274
No	376	236	140	
Yes	24	18	6	

Table S3. Correlation of YTHDF1 protein expression and clinical characteristics (YTHDF1 expressions in the peripheral invasive margin of tumors).

Characteristics	N = 389	YTHDF1 expression		P value
		High (N = 192)	Low (N = 197)	
**Gender**				0.0001
Male	247	140	107	
Female	142	52	90	
**Age (years)**				0.0301
< 60	206	91	115	
≥60	183	101	82	
**Lymphatic meta**				0.0098
No	119	47	72	
Yes	270	145	125	
**Vascular meta**				0.5064
No	327	159	168	
Yes	62	33	29	
**Perineural meta**				0.1159
No	133	73	60	
Yes	256	119	137	
**MSI**				0.1041
0	326	155	171	
1~2	63	37	26	
**TNM 8th**				0.2934
1~2	182	95	87	
3	207	97	110	
**Preexisting_adenoma**				0.3639
No	365	178	187	
Yes	24	14	10	
